# Psychometric properties of stigma and discrimination measurement tools for persons living with HIV: a systematic review using the COSMIN methodology

**DOI:** 10.1186/s13643-024-02535-y

**Published:** 2024-04-27

**Authors:** Yizhu Zhang, Xianxia Yang, Xinru Chai, Shuyu Han, Lili Zhang, Ying Shao, Jianhong Ma, Ke Li, Zhiwen Wang

**Affiliations:** 1 School of Nursing, Peking University, Beijing, 100191 China; 2 School of Public Health, Wuhan University, Wuhan, China; 3 Department of Nursing, Beijing Youan Hospital Affiliated with Capital Medical University, Beijing, 100069 China; 4 Department of Infection, Beijing Youan Hospital Affiliated with Capital Medical University, Beijing, 100069 China; 5 Department of Emergency, Peking University First Hospital, Beijing, 100034 China; 6 Peking University Health Science Centre for Evidence-Based Nursing: A Joanna Briggs Institute Affiliated Group, Beijing, 100191 China

**Keywords:** Acquired Immunodeficiency Syndrome, Patient Reported Outcome Measures, Stigma and Discrimination, Psychometrics, Systematic Review

## Abstract

**Background:**

The development of antiretroviral therapy broadly extends the life expectancy of persons living with HIV (PLHIV). However, stigma and discrimination are still great threat to these individuals and the world's public health care system. Accurate and reproducible measures are prerequisites for robust results. Therefore, it is essential to choose an acceptable measure with satisfactory psychometric properties to assess stigma and discrimination. There has been no systematic review of different stigma and discrimination tools in the field of HIV care. Researchers and clinical practitioners do not have a solid reference for selecting stigma and discrimination measurement tools.

**Methods:**

We systematically searched English and Chinese databases, including PubMed, EMBASE, CINAHL, Web of Science, PsycINFO, ProQuest Dissertations and Theses, The Cochrane Library, CNKI,, and Wanfang, to obtain literature about stigma and discrimination measurement tools that have been developed and applied in the field of HIV. The search period was from 1st January, 1996 to 22nd November 2021. The COnsensus-based Standards for the selection of health Measurement INstruments (COSMIN) guideline (2018 version) was applied to assess the risk of bias for each involved study and summarize the psychometric properties of each tool. The modified version of the Grading of Recommendations Assessment, Development, and, Evaluation (GRADE) method was used to grade the evidence and develop recommendations.

**Results:**

We included 45 studies and 19 PROMs for HIV/AIDS-related stigma and discrimination among PLHIV. All studies had sufficient methodological quality in content validity, structural validity, internal consistency, and the hypothesis testing of structural validity. Limited evidence was found for cross-cultural validity, stability, and criterion validity. No relevant evidence was found concerning measurement error and responsiveness. The Internalized AIDS-related Stigma Scale (IARSS), Internalized HIV Stigma Scale (IHSS), and Wright's HIV stigma scale (WHSS) are recommended for use.

**Conclusions:**

This study recommends three PROMs for different stigma and discrimination scenarios, including IARSS for its good quality and convenience, IHSS for its broader range of items, higher sensitivity, and greater precision, and WHSS for its comprehensive and quick screening. Researchers should also consider the relevance and feasibility of the measurements before putting them into practice.

**Systematic review registration:**

PROSPERO CRD42022308579

**Supplementary Information:**

The online version contains supplementary material available at 10.1186/s13643-024-02535-y.

## Background

Antiretroviral therapy (ART) has reduced HIV-related morbidity, hospitalization, and mortality by 60% to 80% [1], allowing persons living with HIV (PLHIV) to have a near-normal life expectancy [2]. To further control the AIDS epidemic, The Joint United Nations Programme on HIV and AIDS (UNAIDS) proposed a “95-95-95” goal based on the “90-90-90” goals ,but as of 2022, only 5 countries achieved this goal [3]. Furthermore, some researchers advocated a fourth “90” to complement the significance of this goal: 90% of PLHIV who have achieved virologic suppression to obtain a higher quality of life [4]. However, PLHIV still have a much lower quality of life than the public even if they have achieved virologic suppression [5]. Inequality has a significantly impact on the quality of life for PLHIV [6]. Affected by stigmatic attitude, PLHIV are regarded as “HIV tainted” population, possessing a lower position than normal people [7]; and due to discriminating behaviors , PLHIV face more challenges when seeking help from the society [8, 9].

UNAIDS defines HIV-related stigma and discrimination as the unfair treatment of individuals based on established or suspected HIV serological status under equal circumstances [10]. PLHIV are usually excluded by society because they are regarded as homosexuals, injecting drug users, or sex workers [11]. In addition, physical deficits and psychological disorders caused by AIDS progression and treatment can also lead to misunderstandings by the public [12]. Thus, it is not surprising to find that over 50% PLHIV have experienced different kinds of stigma or discrimination [13–15]. A variety of stigma and discrimination is directed against PLHIV, such as negative social attitudes, identity, and beliefs, and imposed violence, rejection, pre-determined blame, and humiliation from others [16, 17]. It hinders HIV testing, reduces PLHIV’s motivation for treatment, decreases treatment adherence, causes social alienation, and severely affects physical and mental health of PLHIV [18, 19]. To cope with this problem, the United Nations convened the fifth High Level Meeting on the Implementation of the Declaration of Commitment on HIV/AIDS in June 2021, with the theme of eliminating inequalities [20]. The latest draft of the Declaration urges for ending stigma and discrimination against key populations. It will be difficult to end the AIDS epidemic without measures to address serious inequalities [21, 22].

The implementation of appropriate Patient Reported Outcome Measures (PROMs) [23] to assess stigma and discrimination is a prerequisite to help PLHIV alleviate the negative effects of stigma and discrimination [24, 25]. There are several measurement tools that have been developed with multiple versions: the Berger HIV Stigma Scale (BHSS) [26], the Kalichman's Internalized AIDS-Related Stigma Scale (IA-RSS) [27], and Wright's HIV stigma scale (WHSS) [28]. As one of the earliest HIV-specific stigma scales, BHSS [26] is the most commonly accepted and used tool. BHSS has been developed into various versions for different measurement settings. IA-RSS [27] contains six items of two dimensions measuring disclosure concerns and negative self-image of PLHIV. The original version of WHSS [28] has 12 items and was developed for Thai youth, while later versions shifted the focus to adult PLHIV [29–32]. Accuracy and reproducibility are the prerequisites of reliable results of PROMs, so the quality of psychometric properties is a critical element to evaluate when selecting PROMs [33, 34]. However, there is an absence of systematic reviews on different kinds of stigma and discrimination instruments in PLHIV across the world, and researchers and clinical practitioners cannot find a reference to select the most appropriate PROMs for their research contexts.

This study aim to conduct a systematic review of stigma and discrimination measurement tools for PLHIV based on COnsensus-based Standards for the selection of health Measurement INstruments (COSMIN) guidelines [23], which will evaluate the psychometric properties of relevant PROMs and provide a comprehensive picture of measurement tools in a research field. Our attempts may be conducive for clinical practitioners and researchers to obtain more reliable data by selecting appropriate instrument on an evidence-based basis, and achieve more significant treatment effect with better intervention timing.

## Methods

### Design

This systematic review is designed based on the COSMIN methodology, and reported according to the Preferred Reporting Items for Systematic Reviews and Meta-Analyses (PRISMA) 2020 (Appendix 1 PRISMA checklist) [35]. We prospectively registered the current review in the PROSPERO database (registration number: CRD42022308579) [36]. Research details was published in previous protocol [37].

### Search strategy

Three steps were followed in the search strategy. First, we conducted primary searches in PubMed using both MeSH terms and free terms to develop search words, and then developed search strategy with relevant search filters by COSMIN [38]. The identified search strategy was confirmed by our research group. Second, we executed the search strategy in PubMed, EMBASE, CINAHL, Web of Science, PsycINFO, ProQuest Dissertations and Theses, The Cochrane Library, CNKI, and Wanfang Data. As ART was first used in 1996 [39], the search period of this study was limited from 1st January, 1996 to 22nd November 2021. Third, we included grey literature through Baidu Scholar and Google Scholar and used the snowball method to manually include literature during screening. Search strategies for all the databases are available in Appendix 2 Searching strategy.

### Eligibility criteria

#### Inclusion criteria

The inclusion criteria were as follows: (a) targeting at adult PLHIV (aged ≥18 years); (b) measuring HIV/AIDS-related stigma and discrimination; (c) focusing on PROMs, including self-report, interview-based, and proxy reports; (d) results covering at least one of the measurement properties required by COSMIN guidelines; and (e) published in either English or Chinese.

#### Exclusion criteria

The exclusion criteria were as follows: (a) full text is not available; (b) duplicate publications; (c) only indirect evidence of psychometric properties was provided in studies.

### Study screening and document selection

We imported all records into NoteExpress V3.X. After removing duplicates, two researchers (Yizhu Zhang & Xianxia Yang) who were trained in evidence-based methodologies independently filtered references first by reading titles, abstracts, and then full texts. If there was any discrepancy, the third researcher (Shuyu Han) wold be consulted. The agreement among researchers at the full-text screening stage was over 70%. Reasons for exclusion of studies at each screening stage were recorded.

### Methodological quality appraisal

Two researchers (Yizhu Zhang & Xianxia Yang) applied the COSMIN Risk of Bias (RoB) Checklist [35] to independently evaluate the methodological quality of the included studies. Then, two researchers cross-checked the evaluation results. Any differences was resolved in consultation with the third researcher (Shuyu Han).

The COSMIN-RoB Checklist consists of 10 dimensions (116 items), which cover PROM development, content validity, construct validity, internal consistency, cross-cultural validity/measurement invariance, reliability, measurement error, criterion validity, hypothesis testing of construct validity, and responsiveness. The options for items are “very good”, “adequate”, “doubtful”, “inadequate”, and “NA (not applicable)”. The assessment of methodological quality was based on the "worst-score counts" principle: the final rating was determined the item with the worst methodological quality in the evaluation dimension.

### Data extraction

Two researchers (Yizhu Zhang & Xianxia Yang) independently extracted and cross-checked the data, which were divided into two parts: study characteristics and PROM characteristics. Study characteristics included author, publication year, PROM’s title, language, country, study design, population characteristics, and year of development/validation. PROM characteristics also included target population, mode of administration, construct/domain, recall period, number of items, response options, range of scores, original language, and theory. If there were missing data from the included studies, the content of the corresponding information extraction would be marked with "-". Any disparities found during cross-checking were discussed by the two researchers and resolved with the third researcher (Shuyu Han).

### Measurement properties quality appraisal

There are nine dimensions in the evaluation criteria of COSMIN [22], including structural validity, internal consistency, reliability, measurement error, hypothesis testing for construct validity, cross-cultural validity/measurement invariance, criterion validity, and responsiveness. Two researchers (Yizhu Zhang & Xianxia Yang) independently extracted the studies' results and evaluated them by the criteria. Each result of the measurement properties was rated as “suffcient (+)”, “insuffcient (-)”, or “indeterminate (?)”. If one study was rated as NA in the methodological quality appraisal, this dimension was not evaluated for measurement properties. If different studies of the same PROM were rated the same, ratings of the measurement properties would be kept the same; if the measurement properties were rated differently, the studies would be divided into subgroups according to the reasons for the inconsistency, such as different languages, populations, or cultural environments. If the reason for the inconsistency could not be found, the attribute would be evaluated as “inconsistent (±)”. When there was no evidence of “sufficient (+)” findings to support the attribute, the attribute would be rated as “uncertain (?)”.

### Summarizing and grading the evidence

Applying the modified Grading of Recommendations Assessment, Development, and, Evaluation (mGRADE) [23], four researchers (Yizhu Zhang, Xianxia Yang, Shuyu Han, and Ke Li) rated the properties of the measurement tools for HIV-related stigma and discrimination in PLHIV based on four downgrading factors: risk of bias, inconsistency, imprecision, and indirectness. Each measurement property would be rated as high, moderate, low, and very low. When information was not extracted, it would be recorded as “NA”. The expert group also took different research scenarios into consideration when grading the quality level of evidence. If there was disagreement in the evaluation, it would be taken to the fifth researcher (Zhiwen Wang) for resolution. Based on the mGRADE results, four researchers used the COSMIN recommendation score to classify them as A (recommended for use), B (have the potential to be recommended), and C (not recommended), and pick the best PROMs.

## Results

### Literature search

**Fig. 1:**
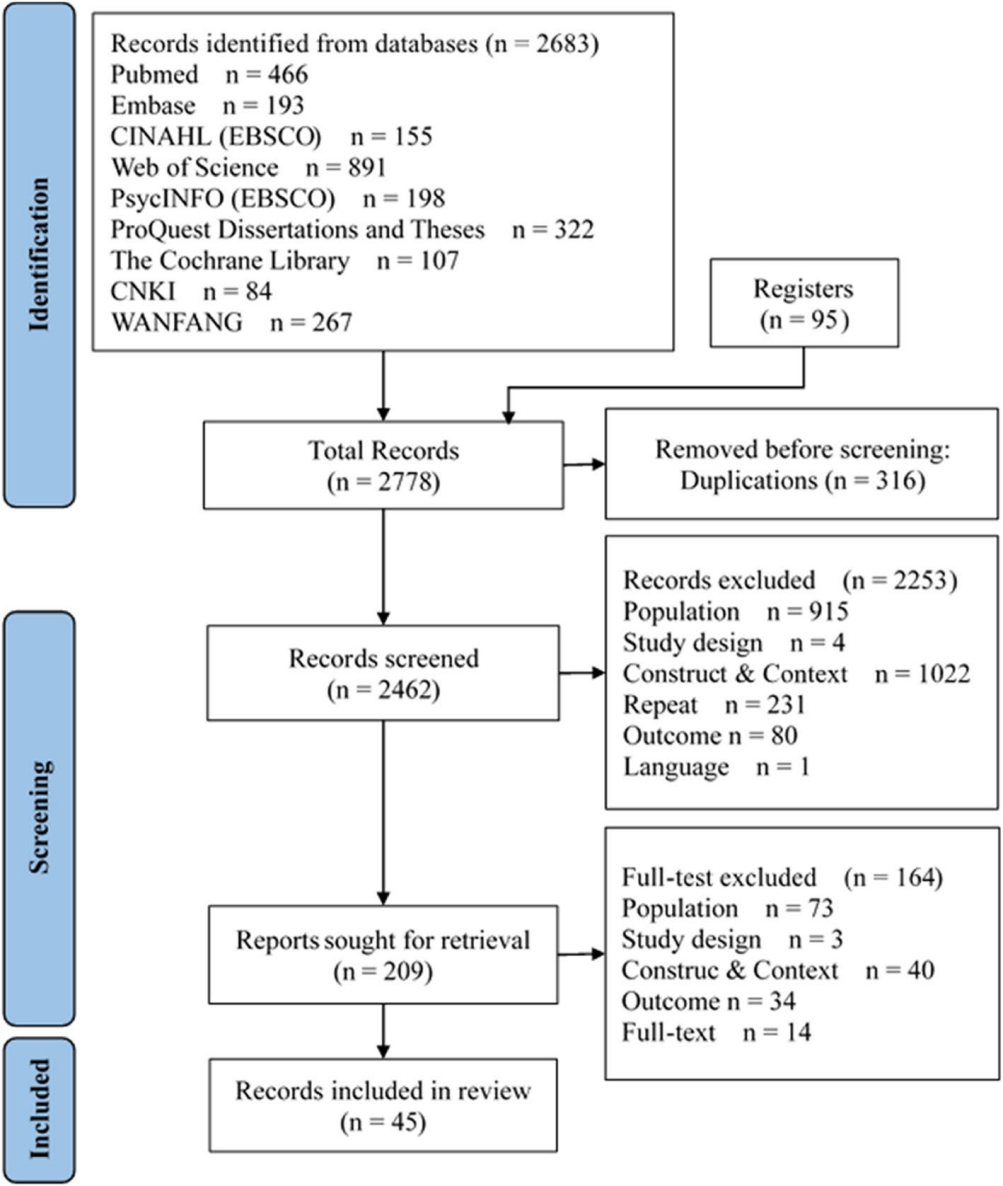
PRISMA 2020 flowchart of the identification and selection of studies

In preliminary searches, 2683 relevant studies were obtained from nine major databases, and 95 additional studies were added manually. A total of 316 duplications were excluded by the NoteExpress automatic check. For the remaining 2462 articles, 2152 were in English and 310 were in Chinese. We excluded 2253 papers by reading the title and abstract and 164 papers by reading the full text. Finally, 45 studies were included covering 19 PROMs. The literature screening process is illustrated in Fig. 1 PRISMA 2020 flowchart of the identification and selection of studies.

### Interpretable description

#### Characteristics of the included studies

Out of 45 included studies, a total of 40 were published in English, and five were published in Chinese between 2000 and 2021. Characteristics of the included studies are shown in Appendix 3 Characteristics of the included studies. Study settings includes the US [26, 27, 29, 40–50], China [51–57], India [58–62], Spain [63–65], and South Africa [27, 66, 67]. With regard to study type, 40 were cross-sectional studies [26, 27, 29–32, 40–42, 44–46, 48–61, 64, 65, 67–77], two were cohort studies [66, 78], two were case-control studies [43, 62], and one was a randomized controlled trial [47]. There were 36,257 participants in these studies, with sample sizes ranging from 25 to 13,183. In addition, some research restricted the target population in females [48, 73], rural residents [40, 48, 60], or those under treatment [46, 61, 73, 74].

#### Characteristics of the included PROMs

The measurement characteristics of the 19 PROMs are shown in Appendix 4 Quality appraisal. Most of them were self-reported [26, 27, 30, 41, 47, 48, 51, 52, 54, 62, 65, 66, 70, 76]. In the structures/measurement domain, only the ATIS is single dimensiona l[47], and 14 PROMs contain internalized stigma [26, 27, 30, 48, 51, 54, 55, 59, 61, 62, 65–67, 70]. Only eight studies reported the recall period [31, 47–49, 52, 64, 66, 78]. The number of items in the 19 PROMs ranged from 4 to 40, with a medium of 17. In the revision of the scale, 12 PROMs applied CTT theory [26, 45, 47, 48, 51, 52, 54, 62, 65, 66, 70, 76], three applied IRT theory [30, 55, 67], and four did not report the method of preparation [27, 41, 59, 61].

### Quality appraisal

#### Methodological quality appraisal

All included studies were methodologically qualified to be evaluated for further study and are shown in Appendix 5 Methodological quality appraisal. In the PROM development, 12 studies were evaluated as inadequate due to the absence of cognitive interviews or another pilot test [29, 42, 46, 52, 53, 55, 58, 60, 67, 69, 73, 74], and 14 were rated as doubtful for only having a quantitative survey and an inadequate number of participants [26, 27, 31, 40, 41, 43, 45, 48, 56, 65, 66, 68, 75, 77]. The most frequent reason for downgrading in content validity was “not tested on an appropriate number of professionals”. All 39 studies were tested for construct validity, where 21 were rated as adequate for only having exploratory factor analysis [26, 31, 43–45, 47–59, 64, 66, 68, 69, 72–74]. Only two studies did not report internal consistency [29, 61]. The rest dimensions are reported by less than half of included scales. Common downgrading reasons are insufficient sample size [42, 43, 49, 68] convenience sampling [41, 46, 50, 58, 59, 61, 63, 64, 67, 69, 71, 78], the statistical methods outside of the COSMIN-RoB Checklist [26, 27, 51, 53, 68, 72], gold standard not an HIV-related stigma and discrimination scale [52–55, 64], and comparison tool's measurement properties were unclear [50]. No relevant evidence regarding measurement error and responsiveness was found in 45 included studies.

#### Quality appraisal of measurement properties

The quality of the measurement properties are shown in Appendix 6 Measurement properties quality appraisal. No findings on measurement error or responsiveness were found in any of the 45 included studies. In structural validity, 12 studies were rated as “+” [29, 30, 32, 46, 60, 63, 65, 67, 68, 70, 71, 78], five studies as “-” [40, 51, 55, 57, 76], and 22 studies were graded as “?” because they did not do it [26, 31, 43–45, 47–50, 52–54, 56, 58, 62, 64, 66, 69, 72–75]. In internal consistency, 31 studies were rated as “+” [26, 27, 30, 32, 40–42, 44, 47–49, 51, 52, 54, 56–59, 62–66, 68–70, 72, 74–76, 78], whereas 12 studies were rated as “-” [31, 43, 45, 46, 50, 53, 55, 60, 67, 71, 73, 77]. Of the 15 studies with reliability tests, six were “+” [56, 60, 64, 75–77], two were “-” [47, 74], and seven were “?” because the ICC was not reported [26, 27, 51, 53, 54, 68, 72].

### Evidence grading and recommendations

Based on the quality assessment results, three PROMs were rated as A level [27, 30, 50], 10 PROMs were B [26, 45, 47, 48, 51, 52, 54, 58, 62, 76], and six PROMs were C [41, 55, 61, 65–67]. The result of the PLHIV stigma scale mGRADE is shown in Appendix 7 Evidence grading and recommendations.

We recommend the IARSS [27], IHSS [50], and WHSS [28]. In the six versions of the IARSS [27, 58, 64, 74, 75, 78], two were rated as high [64, 78] and two were rated as moderate [58, 75] in content validity. Five studies were rated as high [27, 58, 64, 74, 78] and one was rated as moderate [74] in internal consistency. Moreover, five studies conducting hypothesis testing for structural validity were rated as high [27, 58, 64, 74, 78]. All three versions of the IHSS had moderate content validity and high internal consistency [46, 50, 56]. The WHSS has four versions [29,30,32,44, two were rated as high [32, 44] whereas one was rated as moderate [30] in content validity. In addition, two studies were rated as high [30, 32] and one was rated as moderate [43] in internal consistency. Although the BHSS has the most versions [26], no study reported a high internal consistency rating. Compared to the recommended PROMs, its remaining eight measurement properties were reported and rated lower.

## Discussion

To our knowledge, this is the first systematic review to summarize HIV/AIDS-related stigma and discrimination measurement tools for PLHIV. A total of 45 studies on 19 stigma and discrimination measurement tools for PLHIV were included in this systematic review, covering a more comprehensive range of measurement instruments than other reviews in this direction. The findings of our study will provide researchers and practitioners with a quantitative evidence for selecting tools to measure stigma and discrimination in PLHIV and offer new ideas about the direction of future research.

The IARSS [27] has the highest evidence level for psychometric properties among all the included measurement instruments. Although we did not find any systematic review about the psychometric properties and application scenarios of the IARSS, it has been used by hundreds of articles [79], proving investigators’ acknowledgement of its quality. Therefore, our group agreed that the IARSS has good quality and is more convenient. The IHSS [50] is mainly used in qualitative research of stigma [80] as well as measuring the relationship between stigma and depression [81], HIV-positive reports [82], and sexual minorities [83]. Due to its broader range of items, higher sensitivity, and greater precision, the IHSS is suitable to validate the effects of interventions. The WHSS [28] was derived from the BHSS [26] as a simplified version with the same dimensions. As the original version of the WHSS only included adolescents, our study obtained versions that measured adult PLHIV in other languages. As a multidimensional instrument of stigma, the WHSS provides a comprehensive measure of stigma and is suitable as a quick screening tool.

According to the literature results, only a limited amount of research comes from grounded theory and has specific limitations in the target population. Enrolment is mostly in hospitals or specialty clinics, which leaves out PLHIV who are more likely to be experiencing inequality and higher levels of stigma and discrimination. Both of the above would lead to underrepresentation of measurement tools. In recent years, new measurement tools in this field keep emerging, but their interpretability, applicability, and measurement quality do not see significant improvement. If researchers simply develop new tools instead of expanding the scope and improving the quality of existing tools, more research may not be as valuable as it could be. With the development of evidence-based medicine, COSMIN can help us not only in evaluating instruments, but also in making checklists for researchers to develop and validate high-quality measurement tools [84], as well as developing guidelines on how to report measurement tools [85]. More specifically, it can support the development and reporting of PLHIV stigma and discrimination measurement tools.

Several limitations to this study should be noted. First, due to language limitation, our study only included English and Chinese literature, leading to narrowed sample size and bias. Nevertheless, this bias would not affect the evaluation outcome of any measurement tool. Second, PLHIV usually suffer from physical and psychological disruptions, so the intersecting stigma and discrimination of illness, psychological impairment and physical disability would influence the results [86–88]. None of the included literature reported this concern. Finally, though meta-analysis could be a good approach to report this kind of research, the heterogeneity of the results made a meta-analysis infeasible. Therefore, a narrative synthesis was conducted to recapitulate the findings.

## Conclusions

The systematic review included 45 original studies covering 19 HIV/AIDS-related stigma and discrimination measurement tools for PLHIV. Following data extraction, quality appraisal, and mGRADE rating, we recommend three PROMs: a long instrument, the IHSS, and two short instruments, the IARSS and WHSS. At the same time, we suggest that practitioners should thoroughly consider the relevance and usefulness of measurement tools before selecting one. Compared with other studies in this direction, this study contains a more comprehensive inclusion of PROMs. The findings can provide a quantitative basis for the selection of tools to measure HIV/AIDS-related stigma and discrimination for researchers and practitioners and provide a fresh perspective for future research in this field.

### Supplementary Information

**Supplementary Material 1.**

**Supplementary Material 2.**

**Supplementary Material 3.**

**Supplementary Material 4.**

**Supplementary Material 5.**

**Supplementary Material 6.**

**Supplementary Material 7.**

## Data Availability

All data generated or analysed during this study are included in this published article [and its supplementary information files].

## Abbreviations

PLHIV: Persons living with HIV
COSMIN: COnsensus-based Standards for the selection of health Measurement INstruments
GRADE: Grading of Recommendations Assessment, Development, and, Evaluation
ART: Antiretroviral therapy
UNAIDS: Joint United Nations Programme on HIV and AIDS
PROM: Patient Reported Outcome Measures
PRISMA: Preferred Reporting Items for Systematic Reviews and Meta-Analyses
RoB: COSMIN Risk of Bias
mGRADE: modified Grading of Recommendations Assessment, Development, and, Evaluation
BHSS: Berger HIV Stigma Scale
CIBHSS: Berger HIV Stigma Scale for Chronic Illness
HFSS: HIV Felt-Stigma Scale
IARSS: Internalized AIDS-related Stigma Scale
WHSS: Wright’s HIV stigma scale
IHSS: Internalized HIV Stigma Scale
HASIP: HIV/AIDS Stigma Instrument-PLWHA
HRSS: HIV/AIDS Related Stigma Scale
DS: Discrimination Scale
HAFSS: HIV/AIDS Felt-Stigma Scale
IHSS2: Internalized HIV Stigma Scale 2
ARSI: Abuse Related Shame Inventory
HRS: HIV-Related Stigma
EDS: Everyday Discrimination Scale
BC: Brief COPE
VRHRSS: Van Rie HIV/AIDS-Related Stigma Scale
HSPS: HIV/AIDS Stigma Parallel Scale
PSHS: Perceived Stigma HIV/AIDS Scale
ATIS: Internalized Stigma of AIDS Tool
MSPD: Multidimensional Scale of Perceived Discrimination
IHSS3: Internalized HIV Stigma Scale 3
