# Supplementary material for: Psychometric properties of stigma and discrimination measurement tools for persons living with HIV: a systematic review using the COSMIN methodology
Source: Syst Rev. 2024 Apr 27;13:115. doi: 10.1186/s13643-024-02535-y (PMC11055308; doi:10.1186/s13643-024-02535-y)
Supplement: Supplementary file 2 — Supplementary Material 2. [file 13643_2024_2535_MOESM2_ESM.docx]

**PubMed**

Search time：2021-11-22

| **Search** | **Query** | **Items** |
| --- | --- | --- |
| #1 | ("HIV infections"[MeSH Terms] OR "HIV"[MeSH Terms] OR "acquired immunodeficiency syndrome"[MeSH Terms] OR "HIV"[Title/Abstract] OR "hiv infect*"[Title/Abstract] OR "AIDS"[Title/Abstract] OR "PLWHA"[Title/Abstract] OR "PLWH"[Title/Abstract] OR "human immunodeficiency virus"[Title/Abstract] OR ("human immun*"[Title/Abstract] AND "deficiency virus"[Title/Abstract]) OR "acquired immunodeficiency syndrome"[Title/Abstract] OR ("acquired immun*"[Title/Abstract] AND "deficiency syndrome"[Title/Abstract])) | 118,945 |
| #2 | ("social stigma"[MeSH Terms] OR "discrimination, psychological"[MeSH Terms] OR "social discrimination"[MeSH Terms] OR "stigma"[Title/Abstract] OR "discrimination"[Title/Abstract] OR "social distance"[Title/Abstract] OR "social opinion"[Title/Abstract] OR "victimi*"[Title/Abstract] OR "disgrace"[Title/Abstract] OR "stress"[Title/Abstract] OR "prejud*"[Title/Abstract] OR "hostil*"[Title/Abstract] OR "harass*"[Title/Abstract] OR "bully*"[Title/Abstract] OR "unfair treat*"[Title/Abstract] OR "oppress*"[Title/Abstract] OR "rejection"[Title/Abstract]) | 214,161 |
| #3 | (("patient reported outcome measures*"[MeSH Terms] OR "Surveys and Questionnaires"[MeSH Terms] OR "scale*"[Title/Abstract] OR "measur*"[Title/Abstract] OR "assessment"[Title/Abstract] OR "patient reported outcome measur*"[Title/Abstract] OR "prom"[Title/Abstract]) | 1,694,575 |
| #4 | ("instrumentation"[MeSH Subheading] OR "methods"[MeSH Subheading] OR "Comparative Study"[Publication Type] OR "psychometrics"[MeSH Terms] OR "psychometr*"[Title/Abstract] OR "clinimetr*"[Text Word] OR "clinometr*"[Text Word] OR "outcome assessment"[Title/Abstract] OR "outcome measure*"[Text Word] OR "observer variation"[MeSH Terms] OR "observer variation"[Title/Abstract] OR "Health Status Indicators"[MeSH Terms] OR "reproducibility of results"[MeSH Terms] OR "reproducib*"[Title/Abstract] OR "discriminant analysis"[MeSH Terms] OR "reliab*"[Title/Abstract] OR "unreliab*"[Title/Abstract] OR "valid*"[Title/Abstract] OR "coefficient of variation"[Title/Abstract] OR "coefficient"[Title/Abstract] OR "homogeneity"[Title/Abstract] OR "homogeneous"[Title/Abstract] OR "internal consistency"[Title/Abstract] OR ("cronbach*"[Title/Abstract] AND ("alpha"[Title/Abstract] OR "alphas"[Title/Abstract])) OR ("item"[Title/Abstract] AND ("correlation*"[Title/Abstract] OR "selection*"[Title/Abstract] OR "reduction*"[Title/Abstract])) OR "agreement"[Text Word] OR "precision"[Text Word] OR "imprecision"[Text Word] OR "precise values"[Text Word] OR "test-retest"[Title/Abstract] OR ("test"[Title/Abstract] AND "retest"[Title/Abstract]) OR ("reliab*"[Title/Abstract] AND ("test"[Title/Abstract] OR "retest"[Title/Abstract])) OR "stability"[Title/Abstract] OR "interrater"[Title/Abstract] OR "inter-rater"[Title/Abstract] OR "intrarater"[Title/Abstract] OR "intra-rater"[Title/Abstract] OR "intertester"[Title/Abstract] OR "inter-tester"[Title/Abstract] OR "intratester"[Title/Abstract] OR "intra-tester"[Title/Abstract] OR "interobserver"[Title/Abstract] OR "inter-observer"[Title/Abstract] OR "intraobserver"[Title/Abstract] OR "intra-observer"[Title/Abstract] OR "intertechnician"[Title/Abstract] OR "inter-technician"[Title/Abstract] OR "intratechnician"[Title/Abstract] OR "intra-technician"[Title/Abstract] OR "interexaminer"[Title/Abstract] OR "inter-examiner"[Title/Abstract] OR "intraexaminer"[Title/Abstract] OR "intra-examiner"[Title/Abstract] OR "interassay"[Title/Abstract] OR "inter-assay"[Title/Abstract] OR "intraassay"[Title/Abstract] OR "intra-assay"[Title/Abstract] OR "interindividual"[Title/Abstract] OR "inter-individual"[Title/Abstract] OR "intraindividual"[Title/Abstract] OR "intra-individual"[Title/Abstract] OR "interparticipant"[Title/Abstract] OR "inter-participant"[Title/Abstract] OR "intraparticipant"[Title/Abstract] OR "intra-participant"[Title/Abstract] OR "kappa"[Title/Abstract] OR "kappa's"[Title/Abstract] OR "kappas"[Title/Abstract] OR "repeatab*"[Text Word] OR (("replicab*"[Text Word] OR "repeated"[Text Word]) AND ("measure"[Text Word] OR "measures"[Text Word] OR "findings"[Text Word] OR "result"[Text Word] OR "results"[Text Word] OR "test"[Text Word] OR "tests"[Text Word])) OR "generaliza*"[Title/Abstract] OR "generalisa*"[Title/Abstract] OR "concordance"[Title/Abstract] OR ("intraclass"[Title/Abstract] AND "correlation*"[Title/Abstract]) OR "discriminative"[Title/Abstract] OR "known group"[Title/Abstract] OR "factor analysis"[Title/Abstract] OR "factor analyses"[Title/Abstract] OR "factor structure"[Title/Abstract] OR "factor structures"[Title/Abstract] OR "dimension*"[Title/Abstract] OR "subscale*"[Title/Abstract] OR ("multitrait"[Title/Abstract] AND "scaling"[Title/Abstract] AND ("analysis"[Title/Abstract] OR "analyses"[Title/Abstract])) OR "item discriminant"[Title/Abstract] OR "interscale correlation*"[Title/Abstract] OR "error"[Title/Abstract] OR "errors"[Title/Abstract] OR "individual variability"[Title/Abstract] OR "interval variability"[Title/Abstract] OR "rate variability"[Title/Abstract] OR ("variability"[Title/Abstract] AND ("analysis"[Title/Abstract] OR "values"[Title/Abstract])) OR ("uncertainty"[Title/Abstract] AND ("measurement"[Title/Abstract] OR "measuring"[Title/Abstract])) OR "standard error of measurement"[Title/Abstract] OR "sensitiv*"[Title/Abstract] OR "responsive*"[Title/Abstract] OR ("limit"[Title/Abstract] AND "detection"[Title/Abstract]) OR "minimal detectable concentration"[Title/Abstract] OR "interpretab*"[Title/Abstract] OR (("minimal"[Title/Abstract] OR "minimally"[Title/Abstract] OR "clinical"[Title/Abstract] OR "clinically"[Title/Abstract]) AND ("important"[Title/Abstract] OR "significant"[Title/Abstract] OR "detectable"[Title/Abstract]) AND ("change"[Title/Abstract] OR "difference"[Title/Abstract])) OR ("small*"[Title/Abstract] AND ("real"[Title/Abstract] OR "detectable"[Title/Abstract]) AND ("change"[Title/Abstract] OR "difference"[Title/Abstract])) OR "meaningful change"[Title/Abstract] OR "ceiling effect"[Title/Abstract] OR "floor effect"[Title/Abstract] OR "Item response model"[Title/Abstract] OR "IRT"[Title/Abstract] OR "Rasch"[Title/Abstract] OR "Differential item functioning"[Title/Abstract] OR "DIF"[Title/Abstract] OR "computer adaptive testing"[Title/Abstract] OR "item bank"[Title/Abstract] OR "cross-cultural equivalence"[Title/Abstract]) | 2,164,726 |
| #5 | (("delphi technique"[Title] OR "cross-sectional"[Title] OR "biography"[Publication Type] OR "case reports"[Publication Type] OR "comment"[Publication Type] OR "directory"[Publication Type] OR "editorial"[Publication Type] OR "festschrift"[Publication Type] OR "interview"[Publication Type] OR "legislation"[Publication Type] OR "letter"[Publication Type] OR "news"[Publication Type] OR "newspaper article"[Publication Type] OR "patient education handout"[Publication Type] OR "consensus development conference"[Publication Type] OR "consensus development conference, nih"[Publication Type] OR "practice guideline"[Publication Type]) NOT ("animals"[MeSH Terms] NOT "humans"[MeSH Terms])) | 869,896 |
| #6 | ("protocol"[Title] OR "qualitative"[Title] OR "hear*"[Title/Abstract] OR "prevention"[Title/Abstract] OR "Pre-Exposure Prophylaxis"[MeSH Terms] OR "Pre-Exposure Prophylaxis"[Title/Abstract] OR "Prep"[Title/Abstract] OR "intervention"[Title/Abstract] OR "genes"[MeSH Terms] OR "RNA"[MeSH Terms] OR "proteins"[MeSH Terms] OR "experien*"[Title/Abstract] OR "interview*"[Title/Abstract]) | 1,835,480 |
| #7 | (#1 AND #2 AND #3 AND #4 NOT #5) NOT #6  Filters: Full text, Humans, English,, alladult, 1996/1/1:2021/11/22 | 466 |

**EMBASE**

Search time：2021-11-22

| **Search** | **Query** | **Items** |
| --- | --- | --- |
| #1 | ('hiv':ti,ab,kw OR 'aids':ti,ab,kw OR 'plwha':ti,ab,kw OR 'plwh':ti,ab,kw OR 'acquired immunodeficiency syndrome':ti,ab,kw OR 'hiv infect*':ti,ab,kw OR 'human immunodeficiency virus':ti,ab,kw OR ('human immun*':ti,ab,kw AND 'deficiency virus':ti,ab,kw) OR ('acquired immun*':ti,ab,kw AND 'deficiency syndrome':ti,ab,kw)) | 456,357 |
| #2 | (stigma:ti,ab,kw OR discrimination:ti,ab,kw OR 'social distance':ti,ab,kw OR 'social opinion':ti,ab,kw OR victim:ti,ab,kw OR disgrace:ti,ab,kw OR 'physiological stress':ti,ab,kw OR prejudice:ti,ab,kw OR hostility:ti,ab,kw OR harassment:ti,ab,kw OR bullying:ti,ab,kw OR oppression:ti,ab,kw OR rejection:ti,ab,kw) | 321,513 |
| #3 | ('patient reported outcome measur*':ti,ab,kw OR survey*:ti,ab,kw OR questionnaire*:ti,ab,kw OR scale*:ti,ab,kw OR measur*:ti,ab,kw OR assessment*:ti,ab,kw OR prom:ti,ab,kw) | 6,713,259 |
| #4 | (validity:ti,ab,kw OR reliability:ti,ab,kw OR 'internal consistency':ti,ab,kw OR 'internal consistency reliability':ti,ab,kw OR 'measurement invariance':ti,ab,kw OR 'measurement error?':ti,ab,kw OR 'hypotheses testing':ti,ab,kw OR responsiveness:ti,ab,kw) | 443,866 |
| #5 | #1 AND #2 AND #3 AND #4  Lim to (full text and human and adult and english language and py="1996-2021" | 193 |

**CINAHL**

Search time：2021-11-22

| **Search** | **Query** | **Items** |
| --- | --- | --- |
| #1 | SU hiv OR hiv infect* OR AIDS OR PLWHA OR PLWH OR human immunodeficiency virus OR (human immun* AND deficiency virus) OR acquired immunodeficiency syndrome OR (acquired immun* AND deficiency syndrome) | 27,076 |
| #2 | SU stigma OR discrimination OR social distance OR social opinion OR victimi* OR disgrace OR stress OR prejud* OR hostil* OR harass* OR bully* OR unfair treat* OR oppress* OR rejection | 62,642 |
| #3 | SU scale* OR measur* OR assessment* OR patient reported outcome measur* OR PROM OR survey* OR questionnaire* | 542,601 |
| #4 | SU validity OR reliability OR internal consistency OR measurement invariance OR measurement error? OR hypotheses testing OR responsiveness | 51,165 |
| #5 | #1 AND #2 AND #3 AND #4  Limiters - Full Text; Published Date: 19960101-20211122; Human; All Adult; English | 156 |

**Web of Science**

Search time：2021-11-22

| **Search** | **Query** | **Items** |
| --- | --- | --- |
| #1 | TS=(hiv OR hiv infect* OR AIDS OR plwha OR plwh OR human immunodeficiency virus OR (human immun* AND deficiency virus) OR acquired immunodeficiency syndrome OR (acquired immun* AND deficiency syndrome)) | 1,696,771 |
| #2 | TS=(stigma OR discrimination OR “social distance” OR “social opinion” OR victimi* OR disgrace OR stress OR prejud* OR hostil* OR harass* OR bully* OR unfair treat* OR oppress* OR rejection) | 4,212,701 |
| #3 | TS=(scale* OR measur* OR assessment* OR patient reported outcome measur* OR PROM* OR survey* OR questionnaire*) | 23,878,920 |
| #4 | TS=(validity OR reliability OR internal consistency OR measurement invariance OR measurement error？ OR hypotheses testing OR responsiveness) | 3,440,893 |
| #5 | #8 and Articles or Other or Review Articles or Clinical Trial (Document Types) and English (Languages) and Adolescent or Child or Child Preschool or Child Abuse Sexual or Child Development or Child Orphaned or Child Abuse or Child Of Impaired Parents or Child Behavior or Child Development Disorders Pervasive or Child Behavior Disorders or Child Language or Child Rearing or Child Welfare or Animals or Animal Husbandry or Animals Newborn or Animals Wild or Animal Communication or Animal Experimentation or Animal Feed or Animal Nutritional Physiological Phenomena or Animal Welfare or Animals Genetically Modified or Animals Suckling or Anisoles or Dna Methylation or Dna or Dna Bacterial or Dna Polymerase Beta or Dna Topoisomerases Type I or Dna Viral or Rna Messenger or Rna Interference or Rna Small Interfering or Rna Transfer or Rna Viral or Rna Viruses or Gene Expression or Gene Expression Profiling or General Surgery or Genetic Variation or Gene Expression Regulation or Gene Expression Regulation Neoplastic or Gene Expression Regulation Viral or Genetic Testing or Gene Expression Regulation Bacterial or Gene Expression Regulation Developmental or Gene Expression Regulation Enzymologic or Gene Knockdown Techniques or Gene Products Tat or Genes Fos or Genetic Linkage or Genetic Loci or Genetic Markers or Genetic Predisposition To Disease or Genetic Research or Genetic Speciation or Protein Binding or Protein Isoforms or Proteins or Protein Carbonylation or Protein Conformation or Protein Deglycase Dj 1 or Protein Precursors or Protein Processing Post Translational or Protein Structure Tertiary or Protein Synthesis Inhibitors or Hearing Aids or Hearing Loss or Hearing Loss Sensorineural or Heart Rate or Hearing Tests or Hearing or Hearing Loss Unilateral or Heart or Hearing Loss Bilateral or Heart Valve Prosthesis or Hearing Disorders or Hearing Loss Conductive or Heart Injuries or Heart Valve Prosthesis Implantation or Heart Valves or Heart Ventricles or Dental Stress Analysis or Dental Porcelain or Dental Restoration Failure or Dental Prosthesis Design or Dental Materials or Dental Bonding or Denture Partial Fixed or Dental Veneers or Dental Abutments or Dental Enamel or Dental Prosthesis Implant Supported or Dental Implants or Dental Models or Dental Polishing or Dental Prosthesis or Dental Restoration Permanent or Dental Anxiety or Dental Cements or Dentin or Dentin Bonding Agents or Denture Design or Dental Arch or Dental Care or Dental Care For Children or Dental Caries or Dental Devices Home Care or Dental Etching or Dental Implant Abutment Design or Dental Implantation Endosseous or Dental Impression Technique or Dental Marginal Adaptation or Dental Prosthesis Retention or Dental Pulp or Dental Pulp Diseases or Dental Restoration Wear or Dental Technicians or Dentist Patient Relations or Dentistry or Dentists or Denture Precision Attachment or Anti Hiv Agents or Anti Retroviral Agents or Antiretroviral Therapy Highly Active or Antioxidants or Antiviral Agents or Anti Inflammatory Agents or Qualitative Research or Interviews As Topic or Interview Psychological or Intervertebral Disc or Algorithms (Exclude – MeSH Headings) | 1,542 |
| #6 | TI=(protocol OR qualitative OR hear* OR prevention OR pre-exposure prophylaxis OR pre-exposure prophylaxis OR Prep OR intervention OR gene? OR RNA OR DNA OR proteins OR experien* OR interview) | 4,356,342 |
| #7 | #1 AND #2 AND #3 AND #4 AND #5 NOT #6 AND Engineering or Computer Science (Exclude – Research Areas)  Refined by: Publish Date: 1996-01-01 to 2021-11-22 | 891 |

**PsycINFO**

Search time：2021-11-22

| **Search** | **Query** | **Items** |
| --- | --- | --- |
| #1 | SU hiv OR hiv infect* OR AIDS OR PLWHA OR PLWH OR human immunodeficiency virus OR (human immun* AND deficiency virus) OR acquired immunodeficiency syndrome OR (acquired immun* AND deficiency syndrome) | 43,091 |
| #2 | SU stigma OR discrimination OR social distance OR social opinion OR victimi* OR disgrace OR stress OR prejud* OR hostil* OR harass* OR bully* OR unfair treat* OR oppress* OR rejection | 213,822 |
| #3 | SU scale* OR measur* OR assessment* OR patient reported outcome measur* OR PROM OR survey* OR questionnaire* | 874,112 |
| #4 | SU validity OR reliability OR internal consistency OR measurement invariance OR measurement error？ OR hypotheses testing OR responsiveness | 83,253 |
| #5 | #1 AND #2 AND #3 AND #4  Filters applied: Human, English, Aged: 18+ years, from 1996/1/1-2021/11/22. | 198 |

**ProQuest Dissertations and Theses**

Search time：2021-11-22

| **Search** | **Query** | **Items** |
| --- | --- | --- |
| #1 | ti(hiv OR hiv infect* OR AIDS OR PLWHA OR PLWH OR human immunodeficiency virus OR (human immun* AND deficiency virus) OR acquired immunodeficiency syndrome OR (acquired immun* AND deficiency syndrome)) | 120,850 |
| #2 | ab(hiv OR hiv infect* OR AIDS OR PLWHA OR PLWH OR human immunodeficiency virus OR (human immun* AND deficiency virus) OR acquired immunodeficiency syndrome OR (acquired immun* AND deficiency syndrome)) | 218,835 |
| #3 | #1 AND #2 | 303,389 |
| #4 | ti(stigma OR discrimination OR social distance OR social opinion OR victimi* OR disgrace OR stress OR prejud* OR hostil* OR harass* OR bully* OR unfair treat* OR oppress* OR rejection) | 121,813 |
| #5 | ab(stigma OR discrimination OR social distance OR social opinion OR victimi* OR disgrace OR stress OR prejud* OR hostil* OR harass* OR bully* OR unfair treat* OR oppress* OR rejection) | 412,257 |
| #6 | #4 AND #5 | 467,651 |
| #7 | ti(scale* OR measur* OR assessment* OR patient reported outcome measur* OR PROM OR survey* OR questionnaire*) | 312,293 |
| #8 | ab(scale* OR measur* OR assessment* OR patient reported outcome measur* OR PROM OR survey* OR questionnaire*) | 1,794,049 |
| #9 | #7 AND #8 | 1,918,329 |
| #10 | mainsubject(validity OR reliability OR reliability analysis OR measurement errors OR responsiveness) | 26,908 |
| #11 | ti(validity OR reliability OR internal consistency OR measurement invariance OR measurement error？ OR hypotheses testing OR responsiveness) | 27,406 |
| #12 | ab(validity OR reliability OR internal consistency OR measurement invariance OR measurement error？ OR hypotheses testing OR responsiveness) | 162,053 |
| #13 | #10 AND #11 AND #12 | 180,075 |
| #14 | ti(protocol OR qualitative OR hear* OR prevention OR pre-exposure prophylaxis OR pre-exposure prophylaxis OR Prep OR intervention OR genes OR RNA OR proteins OR experien* OR interview) | 808,428 |
| #15 | #3 AND #6 AND #9 AND #13 NOT #14  Refined by: Languages: English, Chinese; Full text; From 1996-01-01 to 2021-11-22; Publication Types: Academic journal, Dissertation; Document Types: Book, Book chapter, article, These. | 322 |

**The Cochrane Library**

Search time：2021-11-22

| **Search** | **Query** | **Items** |
| --- | --- | --- |
| #1 | (hiv OR hiv infect* OR AIDS OR PLWHA OR PLWH OR human immunodeficiency virus OR (human immun* AND deficiency virus) OR acquired immunodeficiency syndrome OR (acquired immun* AND deficiency syndrome)):ti,ab,kw | 46645 |
| #2 | (stigma OR discrimination OR social distance OR social opinion OR victimi* OR disgrace OR stress OR prejud* OR hostil* OR harass* OR bully* OR unfair treat* OR oppress* OR rejection):ti,ab,kw | 91388 |
| #3 | (scale* OR measur* OR assessment* OR patient reported outcome measur* OR PROM OR survey* OR questionnaire*):ti,ab,kw | 781785 |
| #4 | (validity OR reliability OR internal consistency OR measurement invariance OR measurement error？ OR hypotheses testing OR responsiveness):ti,ab,kw | 395203 |
| #5 | (protocol OR qualitative OR hear* OR prevention OR pre-exposure prophylaxis OR pre-exposure prophylaxis OR Prep OR intervention OR genes OR RNA OR proteins OR experien* OR interview):ti,ab,kw | 921850 |
| #6 | #1 AND #2 AND #3 AND #4 NOT #5  with Cochrane Library publication date from 1996-01 to 2021-11 | 107 |

**CNKI**

Search time：2021-11-22

| **Search** | **Query** | **Items** |
| --- | --- | --- |
| #1 | (TKA='AIDS' + 'HIV' + '艾滋病' + '获得性免疫缺陷综合征' + '人类免疫缺陷病毒') | 123,748 |
| #2 | (TKA='歧视' + '病耻' + '污名' + '羞辱') | 55,678 |
| #3 | (TKA='量表' + '问卷' + '患者报告结局') | 1,177,566 |
| #4 | (TKA='信度' + '效度' + '稳定性' + '内部一致性' + '测量误差' + '反应度' + '可解释性' + '假设检验' + '测量不变性') | 2,287,979 |
| #5 | #1 AND #2 AND #3 AND #4  资源范围:中文；同义词扩展；时间范围：发表时间:1996-01-01-2021-11-22；更新时间:不限 | 83 |

**WANFANG DATA**

Search time：2021-11-22

| **Search** | **Query** | **Items** |
| --- | --- | --- |
| #1 | (主题:(AIDS or HIV or 艾滋病 or 获得性免疫缺陷综合征 or 人类免疫缺陷病毒)) | 119947 |
| #2 | (主题:(歧视 or 病耻 or 污名 or 羞辱)) | 58606 |
| #3 | (主题:(量表 or 问卷 or 患者报告结局)) | 1131728 |
| #4 | (主题:(信度 or 效度 or 稳定性 or 内部一致性 or 测量误差 or 反应度 or 可解释性 or 假设检验 or 测量不变性)) | 5130791 |
| #5 | #1 AND #2 AND #3 AND #4  Date:1996-* | 267 |
