# Supplementary material for: Psychometric properties of stigma and discrimination measurement tools for persons living with HIV: a systematic review using the COSMIN methodology
Source: Syst Rev. 2024 Apr 27;13:115. doi: 10.1186/s13643-024-02535-y (PMC11055308; doi:10.1186/s13643-024-02535-y)
Supplement: Supplementary file 3 — Supplementary Material 3. [file 13643_2024_2535_MOESM3_ESM.docx]

**Table 1**

Characteristics of the included studies

| **Author**  **(year)** | **PROM** | **Country** | **PROM language** | **Study design** | **Participates** | **Years of development/**  **validation** |
| --- | --- | --- | --- | --- | --- | --- |
| Apodaca (2015) | BHSS Spanish version | Spain | Spanish | Cross-sectional study | 557 PLWH,  Age: 43.43 ± 8.09, Rang: 18 - 76,  Male = 394, Female = 156, Transsexual = 7 | 2008 - 2009 |
| Berger (2001) | BHSS | USA | English | Cross-sectional study | 318 PLWH,  Age: 37 ± 7.7, Range: 19 - 82,  Male = 250, Female = 60 | 1994.6 - 1995.4 |
| Bunn (2007) | BHSS-32 | USA | English | Cross-sectional study | 157 PLWH,  Age: 43.2 ± 9.0, Range: 19 - 64,  Male > 112, Female < 45 | - |
| Chan (2019) | IARSS Southern India version | India | Tamil, Telugu | Cross-sectional study | 660 PLWH,  Age: 39.0, Rang: 34 - 45,  Male = 388, Female = 268 | 2015.1 - 2016.11 |

**Table 1 *(Continued)***

Characteristics of the included studies

| **Author**  **(year)** | **PROM** | **Country** | **PROM language** | **Study design** | **Participates** | **Years of development/**  **validation** |
| --- | --- | --- | --- | --- | --- | --- |
| Christopoulos  (2019) | IHSS3 | USA | English | Cross-sectional study | 13183 PLWH,  Age: 49, IQR: 39 - 56, 49% ≥ 50,  Cis-Female = 2373, Fender minority = 395, Sexual minority = 8833 | 2016.2 - 2017.11 |
| Cui (2021) | HAFSS | China | Chinese | Cross-sectional study | 410 PLWH,  Age: 39.78 ± 12.70,  Male = 382, Female = 28 | 2020.7 - 2020.12 |
| Emlet (2005) | HASIP-13 | USA | English, Spanish | Case control study | 88 PLWH,  Older: 44, Mean age: 55.6, Range: 50 - 71;  Younger: 44, Mean age: 34, Range: 20 - 39 | 2002 - 2003 |
| Emlet (2007) | BHSS Spanish version 2 | USA | English | Cross-sectional study | 25 PLWH,  Age: 56.1 ± 5.75, Rang: 50 - 72,  Male = 17, Female = 8 | - |

**Table 1 *(Continued)***

Characteristics of the included studies

| **Author**  **(year)** | **PROM** | **Country** | **PROM language** | **Study design** | **Participates** | **Years of development/**  **validation** |
| --- | --- | --- | --- | --- | --- | --- |
| FIFE (2000) | HRSS | India | English | Cross-sectional study | 130 PLWH,  Age: 36.49 ± 8.04, Range: 23 - 67,  Male = 123, Female = 7 | - |
| Franke (2010) | BHSS Spanish version 3 | Peru | Spanish | Cross-sectional study | 130 PLWH,  Age: 30.5, Rang: 26 - 37 | 2005.11 - 2007.3 |
| Garrido (2017) | IARSS Spanish version | Spain | Spanish | Cross-sectional study | 458 PLWH,  Age: 36.6 ± 10.3, Range: 18 - 75,  Male = 404, Female = 50, Other = 4 | - |

**Table 1 *(Continued)***

Characteristics of the included studies

| **Author**  **(year)** | **PROM** | **Country** | **PROM language** | **Study design** | **Participates** | **Years of development/**  **validation** |
| --- | --- | --- | --- | --- | --- | --- |
| Geibel (2020) | IARSS Cambodia, the Dominican Republic, Uganda, Tanzania version | Cambodia, The Dominican Republic, Uganda, Tanzania | - | Cohort study | 3018 PLWH,  Age: Cambodia = 44.9 ± 9.9, the Dominican Republic = 39.1 ± 10.5, Uganda = 36.2 ± 10.9, Tanzania = 33.4 ± 8.45,  Male = Cambodia = 465, the Dominican Republic = 347, Uganda = 132, Tanzania = 0,  Female = Cambodia = 734, the Dominican Republic = 518, Uganda = 234, Tanzania = 257,  Transgender: Cambodia = 8, the Dominican Republic = 26, Uganda = 25, Tanzania = 0 | 2018 - 2019 |
| Han (2019) | EDS Chinese version | China | Chinese | Cross-sectional study | 885 PLWH,  Male = 600, Female = 285 | 2017.4 - 2017.7 |

**Table 1 *(Continued)***

Characteristics of the included studies

| **Author**  **(year)** | **PROM** | **Country** | **PROM language** | **Study design** | **Participates** | **Years of development/**  **validation** |
| --- | --- | --- | --- | --- | --- | --- |
| Huang (2021) | BHSS Myanmar version | Myanmar | Myanmar | Cross-sectional study | 156 PLWH,  Age: 28.92 ± 17.32,  Male = 97, Female = 58, Transgender = 1 | 2020.1 - 2020.5 |
| Jeyaseelan (2013) | BHSS South Indian version | India | Tamil | Cross-sectional study | 250 PLWH,  Male = Urban (Chennai) = 75,  Semi-rural (Vellore) = 49, Age = 36.78 ± 5.21,  Female = Urban (Chennai) = 75,  Semi-rural (Vellore) = 51, Age = 32.92 ± 6.1 | 2007.12 - 2008.7 |
| Jimenez (2010) | HFSS | Puerto Rico | Spanish | Cross-sectional study | 156 PLWH,  Male = 84, Mean ± SD = 45 ± 8.9,  Female = 72, Mean ± SD = 41 ± 8.2 | 2003 - 2008 |

**Table 1 *(Continued)***

Characteristics of the included studies

| **Author**  **(year)** | **PROM** | **Country** | **PROM language** | **Study design** | **Participates** | **Years of development/**  **validation** |
| --- | --- | --- | --- | --- | --- | --- |
| Johnson (2016) | WHSS United States version | USA | English | Cross-sectional study | 110 PLWH,  Age: 46.1 ± 11.7, Range: 20 - 66,  Male = 49, Female = 58, Transgender = 1 | - |
| Kagiura (2020) | WHSS Japanese version | Japan | Japanese | Cross-sectional study | 449 PLWH,  Age: 47.7 ± 10.7,  Male = 410, Female = 19, Transgender = 4 | 2017.8 - 2018.2 |
| Kalan (2013) | HASIP Iranian Version | Iran | Persian | Cross-sectional study | 200 PLWH,  Age: 35.45 ± 8.49, Range: 18 - 60,  Male = 154, Female = 46 | 2012.5 - 2012.12 |

**Table 1 *(Continued)***

Characteristics of the included studies

| **Author**  **(year)** | **PROM** | **Country** | **PROM language** | **Study design** | **Participates** | **Years of development/**  **validation** |
| --- | --- | --- | --- | --- | --- | --- |
| Kalichman (2008) | IARSS | South Africa, Swaziland, USA | Xhosa, English, Afrikaans | Cross-sectional study | 2397 PLWH,  Male = South Africa = 422, Swaziland = 359, USA = 189,  Women: South Africa = 646, Swaziland = 728, USA = 50 | - |
| Kamitani (2018) | WHSS United States version 2 | USA | English | Cross-sectional study | 67 PLWH,  Age: 44.4 ± 9.5,  Male = 52, Female = 7, Transgender = 8 | - |
| Kingori (2013) | HASIP Kenyan version | Kenya | Swahili | Cross-sectional study | 370 PLWH,  Age: 37.06 ± 8.61,  Male = 223, Female = 145 | 2011 |

**Table 1 *(Continued)***

Characteristics of the included studies

| **Author**  **(year)** | **PROM** | **Country** | **PROM language** | **Study design** | **Participates** | **Years of development/**  **validation** |
| --- | --- | --- | --- | --- | --- | --- |
| Kipp (2015) | VRHRSS | USA | English | Cross-sectional study | 85 PLWH,  Age: 45.0,  Male = 63, Female = 22 | 2011.11 - 2012.1 |
| Li (2010) | BHSS Chinese version | China | Chinese | Cross-sectional study | 578 PLWH,  Age: 36.2 ± 8.8,  Male = 361, Female = 226 | 2008.6 |
| Li (2010) | HRSS and DS | China | Chinese | Cross-sectional study | 307 PLWH,  Age: 38.97 ± 8.81, Range: 18 - 78,  Male = 223, Female = 84 | 2009.4 - 2009.6 |
| Lindberg (2014) | BHSS Swedish version | Sweden | Swedish, English | Cross-sectional study | 194 PLWH,  Age: 48.8 ± 11.7, Range: 19 - 83,  Male = 109, Female = 85 | 2013.3 - 2013.9 |

**Table 1 *(Continued)***

Characteristics of the included studies

| **Author**  **(year)** | **PROM** | **Country** | **PROM language** | **Study design** | **Participates** | **Years of development/**  **validation** |
| --- | --- | --- | --- | --- | --- | --- |
| Luz (2020) | BHSS-12 Brazilian version | Brazilian | Brazilian, Portuguese | Cross-sectional study | 2102 PLWH,  Age range: 25 - 35, Age: Grindr = 38.6 ± 10.1, Social media = 43.7 ± 12.3, Hornet = 37.8 ± 9.9,  Cisgender men: Grindr = 114, Social media = 89, Hornet = 1794,  Cisgender women: Grindr = 0, Social media = 63, Hornet = 0,  Transgender: Grindr = 0, Social media = 0, Hornet = 30 | 2019.10 - 2020.3 |
| Martin (2011) | IHSS Spanish and English version | USA | English, Spanish | Cross-sectional study | 269 PLWH,  Age: 47.5 ± 10.2, Range: 19 - 73,  Male = 211, Female = 57 | - |

**Table 1 *(Continued)***

Characteristics of the included studies

| **Author**  **(year)** | **PROM** | **Country** | **PROM language** | **Study design** | **Participates** | **Years of development/**  **validation** |
| --- | --- | --- | --- | --- | --- | --- |
| Molero (2013) | MSPD | Spain | - | Cross-sectional study | 134 PLWH | 2010 |
| Neufeld (2012) | HIV and ARSI | USA | English | Randomized, controlled trial | 271 PLWH,  Age: 18 - 24 = 11.1%, 25 - 34 = 46.2%, 35 - 44 = 42.7%,  Male = 137, Female = 130, Transgender = 4 | 2002.3 - 2004.1 |
| Öztürk (2020) | IARSS Turkish version | Turkey | Turkish | Cross-sectional study | 60 PLWH,  Age: 41.0 ± 12.09,  Male = 54, Female = 6 | 2018.11 - 2019.5 |
| Phillips (2011) | ATIS | USA | English | Cross-sectional study | 255 PLWH | - |
| Pourmarzi (2015) | HRSS Persian version | Iran | Persian | Cross-sectional study | 80 PLWH,  Age: 33.01 ± 7.99,  Male = 45, Female = 33 | 2014.9 - 2015.1 |

**Table 1 *(Continued)***

Characteristics of the included studies

| **Author**  **(year)** | **PROM** | **Country** | **PROM language** | **Study design** | **Participates** | **Years of development/**  **validation** |
| --- | --- | --- | --- | --- | --- | --- |
| Ranjit (2021) | WHSS Spanish version | Peru | Spanish | Cross-sectional study | 359 PLWH,  Age: 34.0 ± 8.11,  Transgender = 48 | - |
| Rao (2016) | CIBHSS | USA | American | Cross-sectional study | 62 PLWH,  Age range: 23 - 68,  Male = 38, Female = 23, Transgender = 1 | - |
| Reinius (2017) | BHSS-12 Swedish version | Sweden | Swedish | Cross-sectional study | 880 PLWH,  Age: 47.9, Range: 18 - 82,  Male = 651, Female = 229 | 2013.12 - 2014.8 |
| Sayles (2008) | IHSS | USA | English | Cross-sectional study | 202 PLWH,  Age: 43.0, Range: 18 - 76 | 2007.1 - 2007.5 |
| Stangl (2019) | IHSS2 | Zambia, South Africa | Local language | Cohort study | 4053 PLWH,  Male = 477, Female = 3576 | 2013.11 - 2015.3 |

**Table 1 *(Continued)***

Characteristics of the included studies

| **Author**  **(year)** | **PROM** | **Country** | **PROM language** | **Study design** | **Participates** | **Years of development/**  **validation** |
| --- | --- | --- | --- | --- | --- | --- |
| Steward (2008) | HRS | India | Kannada, Telugu, Tamil, English | Cross-sectional study | 229 PLWH,  Age: 37.6, Range: 23 - 74,  Male = 159, Female = 70 | - |
| Su et al. (2015) | BC-PLWH Chinese version | China | Chinese | Cross-sectional study | 258 PLWH,  Age: 18 - 29 = 22.9%, 30 - 39 = 53.1%, 40 - 60 = 24%  Male = 190, Female = 68 | 2007.9 - 2008.1 |
| Tsai (2013) | IARSS Uganda version | Uganda | Runyankole | Cross-sectional study | 456 PLWH,  Age: 35.0, Rang: 29 - 40,  Male = 139, Female = 317 | - |
| Visser (2008) | PSHS African version | South Africa | Sepedi, Setswana, Isizulu | Cross-sectional study | 317 PLWH,  Age: 26.5, Range: 16 - 42,  Male = 171, Female = 146 | - |

**Table 1 *(Continued)***

Characteristics of the included studies

| **Author**  **(year)** | **PROM** | **Country** | **PROM language** | **Study design** | **Participates** | **Years of development/**  **validation** |
| --- | --- | --- | --- | --- | --- | --- |
| Xu (2018) | IHSS Chinese version | China | Chinese | Cross-sectional study | 204 PLWH,  Age: 31.75 ± 8.98, Range: 19 - 61,  Male = 180, Female = 24 | 2017.8 - 2017.9 |
| Yu (2017) | BHSS Chinese version 2 | China (Taiwan) | Chinese | Cross-sectional study | 540 PLWH,  Age: 29.78 ± 7.67,  Male = 537, Female = 3 | 2015.1 - 2015.11 |
| Zelaya (2012) | HSPS | India | Indian | Case control study | 188 PLWH,  Age range: 45.0,  Male = 88, Female = 100, Transgender = 12 | 2007 |

BHSS, Berger HIV Stigma Scale; CIBHSS, Berger HIV Stigma Scale for Chronic Illness; HFSS, HIV Felt-Stigma Scale; IARSS, Internalized AIDS-related Stigma Scale; WHSS, Wright's HIV stigma scale; IHSS, Internalized HIV Stigma Scale; HASIP, HIV/AIDS Stigma Instrument-PLWHA; HRSS, HIV/AIDS Related Stigma Scale; DS, Discrimination Scale; HAFSS, HIV/AIDS Felt-Stigma Scale; IHSS2, Internalized HIV Stigma Scale 2; ARSI, Abuse Related Shame Inventory; HRS, HIV-Related Stigma; EDS, Everyday Discrimination Scale; BC, Brief COPE; VRHRSS, Van Rie HIV/AIDS-Related Stigma Scale; HSPS, HIV/AIDS Stigma Parallel Scale; PSHS, Perceived Stigma HIV/AIDS Scale; ATIS, Internalized Stigma of AIDS Tool; MSPD, Multidimensional Scale of Perceived Discrimination; IHSS3, Internalized HIV Stigma Scale 3
