# Supplementary material for: Psychometric properties of stigma and discrimination measurement tools for persons living with HIV: a systematic review using the COSMIN methodology
Source: Syst Rev. 2024 Apr 27;13:115. doi: 10.1186/s13643-024-02535-y (PMC11055308; doi:10.1186/s13643-024-02535-y)
Supplement: Supplementary file 4 — Supplementary Material 4. [file 13643_2024_2535_MOESM4_ESM.docx]

**Table 2**

Characteristics of the included PROMs

| **Author**  **(year)** | **PROM** | **Target population** | **Mode of adminis-tration** | **Construct/Domain** | **Recall period** | **Number of items** | **Response options** | **Range of scores** | **Original language** | **Theory** |
| --- | --- | --- | --- | --- | --- | --- | --- | --- | --- | --- |
| Apodaca (2015) | BHSS Spanish version | PLWH,  Age ≥ 18 | Self-  report | Personalized stigma,  disclosure concerns,  negative self-image,  concern with public attitudes | - | 30 | 4-point Likert-type response format | 30 - 120 | Chinese | IRT |
| Berger (2001) | BHSS | PLWH,  Age ≥ 18 | Self-  report | Personalized stigma,  disclosure concerns,  negative self-image,  concern with public attitudes | - | 40 | 4-point Likert-type response format | 40 - 160 | English | CTT |

**Table 2 *(Continued)***

Quality appraisal

| **Author**  **(year)** | **PROM** | **Target population** | **Mode of adminis-tration** | **Construct/Domain** | **Recall period** | **Number of items** | **Response options** | **Range of scores** | **Original language** | **Theory** |
| --- | --- | --- | --- | --- | --- | --- | --- | --- | --- | --- |
| Bunn (2007) | BHSS-32 | Rural PLWH | Interview-based | Enacted stigma,  disclosure concerns,  negative self-image,  concern with public attitudes | - | 32 | 4-point Likert-type response format | 32 - 128 | English | IRT |
| Chan (2019) | IARSS Southern India version | PLWH,  Age ≥ 18 | Interview-based | Self-hatred,  fears of disclosure | - | 6 | Binary response | 0 - 6 | Xhosa,  English,  Afrikaans | CTT |
| Christopoulos  (2019) | IHSS3 | PLWH,  Concurrent viremia | Self-  report | - | - | 4 | 4-point Likert-type response format | 4 - 16 | English | - |

**Table 2 *(Continued)***

Quality appraisal

| **Author**  **(year)** | **PROM** | **Target population** | **Mode of adminis-tration** | **Construct/Domain** | **Recall period** | **Number of items** | **Response options** | **Range of scores** | **Original language** | **Theory** |
| --- | --- | --- | --- | --- | --- | --- | --- | --- | --- | --- |
| Cui (2021) | HAFSS | PLWH,  Age ≥ 18 | Self-  report | External discrimination,  internal discrimination | - | 12 | Frequency of stigma: 4-point Likert-type  Impact of stigma:  5-point Likert-type response format  Stigma experience: binary response | - | Chinese | CTT |

**Table 2 *(Continued)***

Quality appraisal

| **Author**  **(year)** | **PROM** | **Target population** | **Mode of adminis-tration** | **Construct/Domain** | **Recall period** | **Number of items** | **Response options** | **Range of scores** | **Original language** | **Theory** |
| --- | --- | --- | --- | --- | --- | --- | --- | --- | --- | --- |
| Emlet (2005) | HASIP-13 | PLWH,  Age: 20 - 39;  Age ≥ 50 | Interview-based | Distancing,  blaming,  discrimination | - | 13 | 4-point Likert-type response format | 13 - 52 | English | CTT |
| Emlet (2007) | BHSS Spanish version 2 | PLWH,  Age ≥ 50 | Interview-based | Personalized stigma,  disclosure concerns,  negative self-image,  public attitudes | - | 40 | 4-point Likert-type response format | 40 - 160 | English | - |
| FIFE (2000) | HRSS | - | Interview-based | Social rejection,  internalized shame,  social isolation,  financial insecurity | - | 24 | 4-point Likert-type response format | 24 - 96 | English | - |

**Table 2 *(Continued)***

Quality appraisal

| **Author**  **(year)** | **PROM** | **Target population** | **Mode of administration** | **Construct/Domain** | **Recall period** | **Number of items** | **Response options** | **Range of scores** | **Original language** | **Theory** |
| --- | --- | --- | --- | --- | --- | --- | --- | --- | --- | --- |
| Franke (2010) | BHSS Spanish version 3 | PLWH,  Living in poverty,  Women & tuberculosis disease enroll priority,  ART ≥ 12 months | Interview-based | Personalized stigma,  disclosure concerns,  negative self-image,  concern with public attitudes | - | 21 | 4-point Likert-type response format | 21 - 84 | English | CTT |
| Garrido (2017) | IARSS *Spanish version* | PLWH,  Age ≥ 18 | Self-  report | Disclosure concerns,  negative self-image | During the last month | 10 | 5-point Likert-type response format | 10 - 50 | Xhosa,  English,  Afrikaans | CTT |

**Table 2 *(Continued)***

Quality appraisal

| **Author**  **(year)** | **PROM** | **Target population** | **Mode of adminis-tration** | **Construct/Domain** | **Recall period** | **Number of items** | **Response options** | **Range of scores** | **Original language** | **Theory** |
| --- | --- | --- | --- | --- | --- | --- | --- | --- | --- | --- |
| Geibel (2020) | IARSS *Cambodia, the Dominican Republic, Uganda, Tanzania version* | PLWH | Interview-based | Internalized stigma | In the past 12 months | 6 | Binary response | 0 - 6 | Xhosa,  English,  Afrikaans | CTT |
| Han (2019) | EDS Chinese version | PLWH | Self-  report | - | In the past 12 months | 10 | 4-point Likert-type response format | 10 - 40 | English | CTT |

**Table 2 *(Continued)***

Quality appraisal

| **Author**  **(year)** | **PROM** | **Target population** | **Mode of adminis-tration** | **Construct/Domain** | **Recall period** | **Number of items** | **Response options** | **Range of scores** | **Original language** | **Theory** |
| --- | --- | --- | --- | --- | --- | --- | --- | --- | --- | --- |
| Huang (2021) | BHSS Myanmar version | PLWH,  Age ≥ 18,  Live in Myanmar | Self-  report | Personalized stigma,  disclosure concerns,  negative self-image,  concern with public attitudes,  religious concerns | - | 35 | 4-point Likert-type response format | 35 - 140 | English | IRT |
| Jeyaseelan (2013) | BHSS South Indian version | PLWH,  Age: 18 - 49 | Interview-based | Personalized stigma,  disclosure concerns,  negative self-image,  public attitudes | - | 25 | 4-point Likert-type response format | 25 - 100 | English | CTT |

**Table 2 *(Continued)***

Quality appraisal

| **Author**  **(year)** | **PROM** | **Target population** | **Mode of adminis-tration** | **Construct/Domain** | **Recall period** | **Number of items** | **Response options** | **Range of scores** | **Original language** | **Theory** |
| --- | --- | --- | --- | --- | --- | --- | --- | --- | --- | --- |
| Jimenez (2010) | HFSS | PLWH,  Age ≥ 18 | Self-  report | Personalized stigma,  disclosure concerns,  negative self-image,  concern with public attitudes | - | 17 | 4 Likert-type scale | 0 - 51 | English | CTT |
| Johnson (2016) | WHSS *United States version* | African American PLWH,  Age ≥ 18 | Self-  report | Social stigma,  self-stigma | - | 10 | 5-point Likert-type response format | 10 - 50 | Thai | IRT |
| Kagiura (2020) | WHSS Japanese version | Japanese PLWH,  Age ≥ 20 | Self-  report | Personalized stigma,  disclosure concerns,  public attitudes,  negative self-image | - | 10 | 5-point Likert-type response format | 10 - 50 | Thai | IRT |

**Table 2 *(Continued)***

Quality appraisal

| **Author**  **(year)** | **PROM** | **Target population** | **Mode of adminis-tration** | **Construct/Domain** | **Recall period** | **Number of items** | **Response options** | **Range of scores** | **Original language** | **Theory** |
| --- | --- | --- | --- | --- | --- | --- | --- | --- | --- | --- |
| Kalan (2013) | HASIP Iranian Version | PLWH,  Age ≥ 18 | Self-  report | Distancing and blaming,  discrimination,  fear | - | 16 | 4-point Likert-type response format | 16 - 64 | Persian | CTT |
| Kalichman (2008) | IARSS | PLWH | Self-  report | Self-defacing beliefs,  negative perceptions | - | 6 | Binary response | 0 - 6 | English | - |
| Kamitani (2018) | WHSS United States version 2 | Self-identify as Asian PLWH,  Age ≥ 18, Speak and read in English. | Self-  report | Personalized stigma,  negative self-image,  public attitudes | - | 13 | 5-point Likert-type response format | 13 - 65 | Thai | CTT |

**Table 2 *(Continued)***

Quality appraisal

| **Author**  **(year)** | **PROM** | **Target population** | **Mode of adminis-tration** | **Construct/Domain** | **Recall period** | **Number of items** | **Response options** | **Range of scores** | **Original language** | **Theory** |
| --- | --- | --- | --- | --- | --- | --- | --- | --- | --- | --- |
| Kingori (2013) | HASIP Kenyan version | PLWH,  Age ≥ 18 | Self-  report | Public attitudes,  ostracize,  discriminate,  personal life disrupted | During the last month | 10 | 5-point Likert-type response format | 18 - 90 | English | CTT |
| Kipp (2015) | VRHRSS | PLWH,  Age ≥ 18 | Interview-based | Loss of social relationships,  managing HIV concealment,  community stigma | - | 15 | 4-point Likert-type response format | 15 - 60 | India | CTT |
| Li (2010) | BHSS *Chinese version* | PLWH,  Age ≥ 18 | Proxy report | Negative self-image,  disclosure concerns,  PLWHA exclusion,  disclosure hurt | - | 22 | Binary response | 22 - 44 | English | CTT |

**Table 2 *(Continued)***

Quality appraisal

| **Author**  **(year)** | **PROM** | **Target population** | **Mode of adminis-tration** | **Construct/Domain** | **Recall period** | **Number of items** | **Response options** | **Range of scores** | **Original language** | **Theory** |
| --- | --- | --- | --- | --- | --- | --- | --- | --- | --- | --- |
| Li (2010) | HRSS and DS | PLWH,  Age ≥ 18 | Self-  report | Disclosure concerns,  internalized stigma,  public rejection,  family stigma,  healthcare providers’ discrimination | - | 34 | 5-point Likert-type response format | 34 - 170 | Chinese | CTT |
| Lindberg (2014) | BHSS Swedish version | PLWH,  Age ≥ 18 | Self-  report | Internalized stigma | - | 39 | 4-point Likert-type response alternatives | 39 - 156 | English | CTT |

**Table 2 *(Continued)***

Quality appraisal

| **Author**  **(year)** | **PROM** | **Target population** | **Mode of adminis-tration** | **Construct/Domain** | **Recall period** | **Number of items** | **Response options** | **Range of scores** | **Original language** | **Theory** |
| --- | --- | --- | --- | --- | --- | --- | --- | --- | --- | --- |
| Luz (2020) | BHSS-12 Brazilian version | Self-  report of HIV infection,  Age ≥ 18,  Live in Brazil | Self-  report | Personalized stigma,  disclosure concerns,  concern with public attitudes,  negative self-image | - | 12 | 4-point Likert-type response format | 12 - 48 | Swedish | CTT |
| Martin (2011) | IHSS Spanish and English version | PLWH,  Age ≥ 18,  Medication ≥ 1 month | Interview-based | Blame and stereotypes,  fear of contagion,  disclosure of stigmatized role,  social relationships | - | 28 | 5-point Likert-type response format | 0 - 100 | English | CTT |

**Table 2 *(Continued)***

Quality appraisal

| **Author**  **(year)** | **PROM** | **Target population** | **Mode of adminis-tration** | **Construct/Domain** | **Recall period** | **Number of items** | **Response options** | **Range of scores** | **Original language** | **Theory** |
| --- | --- | --- | --- | --- | --- | --- | --- | --- | --- | --- |
| Molero (2013) | MSPD | - | Self-  report | Blatant group discrimination,  subtle group discrimination,  blatant individual discrimination,  subtle individual discrimination | - | 20 | 5-point Likert-type response format | 20 - 100 | English | CTT |

**Table 2 *(Continued)***

Quality appraisal

| **Author**  **(year)** | **PROM** | **Target population** | **Mode of adminis-tration** | **Construct/Domain** | **Recall period** | **Number of items** | **Response options** | **Range of scores** | **Original language** | **Theory** |
| --- | --- | --- | --- | --- | --- | --- | --- | --- | --- | --- |
| Neufeld (2012) | HIV and ARSI | PLWH,  Age ≥ 18,  Experienced sexual abuse by someone at least 5 years older than the participant when he < age 18 | Self-  report | HIV-related shame,  impact of HIV related shame on behavior,  sexual abuse-related shame,  shame interaction effects | During the last month | 31 | 5-point Likert-type response format | 0 - 124 | English | CTT |

**Table 2 *(Continued)***

Quality appraisal

| **Author**  **(year)** | **PROM** | **Target population** | **Mode of adminis-tration** | **Construct/Domain** | **Recall period** | **Number of items** | **Response options** | **Range of scores** | **Original language** | **Theory** |
| --- | --- | --- | --- | --- | --- | --- | --- | --- | --- | --- |
| Öztürk (2020) | IARSS Turkish version | PLWH,  Age ≥ 18,  Literate in Turkish | Self-  report | Internalized stigma | - | 6 | Binary response | 0 - 6 | Xhosa,  English,  Afrikaans | CTT |
| Phillips (2011) | ATIS | PLWH women,  Live in the rural southeastern United States | Self-  report | Internalized stigma | Since you were diagnosed | 10 | 5-point Likert-type response format | 10 - 50 | English | CTT |

**Table 2 *(Continued)***

Quality appraisal

| **Author**  **(year)** | **PROM** | **Target population** | **Mode of adminis-tration** | **Construct/Domain** | **Recall period** | **Number of items** | **Response options** | **Range of scores** | **Original language** | **Theory** |
| --- | --- | --- | --- | --- | --- | --- | --- | --- | --- | --- |
| Pourmarzi (2015) | HRSS Persian version | PLWH,  Age ≥ 18 | Self-  report | Social rejection,  negative self-worth,  perceived interpersonal insecurity,  financial insecurity,  discretionary disclosure | - | 20 | 5-point Likert-type response format | 20 - 100 | English | - |
| Ranjit (2021) | WHSS Spanish version | PLWH,  MSM or TGW  Age: 18 - 65 | Self-  report | Internalized stigma,  enacted stigma,  anticipated stigma | - | 8 | 4-point Likert-type response format | 8 - 32 | Thai | IRT |

**Table 2 *(Continued)***

Quality appraisal

| **Author**  **(year)** | **PROM** | **Target population** | **Mode of adminis-tration** | **Construct/Domain** | **Recall period** | **Number of items** | **Response options** | **Range of scores** | **Original language** | **Theory** |
| --- | --- | --- | --- | --- | --- | --- | --- | --- | --- | --- |
| Rao (2016) | CIBHSS | African American PLWH,  Age ≥ 18 | Self-  report | Enacted stigma,  internalized stigma | Lately | 14 | 5-point Likert-type response format | 14 - 70 | English | CTT |
| Reinius (2017) | BHSS-12 Swedish version | PLWH,  Age ≥ 18,  Live in Sweden | Self-  report | Personalized stigma,  disclosure concerns,  concern with public attitudes,  negative self-image | - | 12 | 4-point Likert-type response format | 12 - 48 | English | IRT |
| Sayles (2008) | IHSS | PLWH,  Age ≥ 18 | Self-  report | Blame and stereotypes of HIV,  fear of contagion,  disclosure,  social relationships | - | 28 | 5-point Likert-type response format | 0 - 100 | English | CTT |

**Table 2 *(Continued)***

Quality appraisal

| **Author**  **(year)** | **PROM** | **Target population** | **Mode of adminis-tration** | **Construct/Domain** | **Recall period** | **Number of items** | **Response options** | **Range of scores** | **Original language** | **Theory** |
| --- | --- | --- | --- | --- | --- | --- | --- | --- | --- | --- |
| Stangl (2019) | IHSS2 | PLWH,  Age ≥ 18 | Self-  report | Internalized stigma,  experienced stigma in the community or the healthcare setting | In the past 12 months | 11 | 4-point Likert-type response format | 0 - 33 | Local language | CTT |
| Steward (2008) | HRS | PLWH,  Age ≥ 18,  ART ≥ 1 month | Interview-based | Enacted stigma,  vicarious stigma,  felt normative stigma,  internalized stigma | - | 40 | Enacted Stigma:  binary response  Vicarious Stigma:  4-point Likert-type response format | 0 - 120 | India | - |

**Table 2 *(Continued)***

Quality appraisal

| **Author**  **(year)** | **PROM** | **Target population** | **Mode of adminis-tration** | **Construct/Domain** | **Recall period** | **Number of items** | **Response options** | **Range of scores** | **Original language** | **Theory** |
| --- | --- | --- | --- | --- | --- | --- | --- | --- | --- | --- |
|  |  |  |  |  |  |  | Felt Normative Stigma:  4-point Likert-type response format  Internalized Stigma:  4-point Likert-type response format |  |  |  |

**Table 2 *(Continued)***

Quality appraisal

| **Author**  **(year)** | **PROM** | **Target population** | **Mode of adminis-tration** | **Construct/Domain** | **Recall period** | **Number of items** | **Response options** | **Range of scores** | **Original language** | **Theory** |
| --- | --- | --- | --- | --- | --- | --- | --- | --- | --- | --- |
| Su et al. (2015) | BC-PLWH Chinese version | Chinese PLWH,  Age: 18 - 60 | Interview-based | Self-distraction,  active coping,  denial, substance use,  emotional support,  instrumental support,  behavioral disengagement,  venting,  positive reframing,  planning, humor,  acceptance,  religion,  self-blame | - | 28 | 4-point Likert-type response format | 28 - 112 | English | IRT |

**Table 2 *(Continued)***

Quality appraisal

| **Author**  **(year)** | **PROM** | **Target population** | **Mode of adminis-tration** | **Construct/Domain** | **Recall period** | **Number of items** | **Response options** | **Range of scores** | **Original language** | **Theory** |
| --- | --- | --- | --- | --- | --- | --- | --- | --- | --- | --- |
| Tsai (2013) | IARSS Uganda version | PLWH,  Age ≥ 18,  Newly initiating ART | Interview-based | Internalized stigma | - | 6 | Binary response | 6 - 12 | Xhosa,  English,  Afrikaans | CTT |
| Visser (2008) | PSHS *African version* | Pregnant PLWH | Interview-based | Internalized stigma,  attributed stigma | - | 17 | Binary response | 0 - 17 | English | IRT |
| Xu (2018) | IHSS Chinese version | PLWH | Self-  report | Stereotypes,  disclosure concerns,  social relationships,  self-acceptance | - | 28 | 5-point Likert-type response format | 28 - 140 | English | CTT |

**Table 2 *(Continued)***

Quality appraisal

| **Author**  **(year)** | **PROM** | **Target population** | **Mode of adminis-tration** | **Construct/Domain** | **Recall period** | **Number of items** | **Response options** | **Range of scores** | **Original language** | **Theory** |
| --- | --- | --- | --- | --- | --- | --- | --- | --- | --- | --- |
| Yu (2017) | BHSS Chinese version 2 | PLWH,  Age ≥ 20 | Self-  report | Personalized stigma,  disclosure concerns,  negative self-image,  concern with public attitudes | - | 18 | 4-point Likert-type response format | 18 - 72 | English | IRT |
| Zelaya (2012) | HSPS | PLWH,  Age: 18 - 40 | Self-  report | Internal stigma,  experienced stigma,  perceived stigma | - | 22 | 4-point Likert-type response format | 22 - 88 | India | CTT |
