# Supplementary material for: Psychometric properties of stigma and discrimination measurement tools for persons living with HIV: a systematic review using the COSMIN methodology
Source: Syst Rev. 2024 Apr 27;13:115. doi: 10.1186/s13643-024-02535-y (PMC11055308; doi:10.1186/s13643-024-02535-y)
Supplement: Supplementary file 5 — Supplementary Material 5. [file 13643_2024_2535_MOESM5_ESM.docx]

**Table 3**

Methodological quality appraisal

| **Author**  **(Year)** | **PROM** | **PROM development** | **Content validity** | **Construct validity** | **Internal consistency** | **Cross‐cultural validity/**  **measurement invariance** | **Reliability** | **Measure-ment error** | **Criterion validity** | **Hypothesis testing for construct validity** | **Respon-siveness** |
| --- | --- | --- | --- | --- | --- | --- | --- | --- | --- | --- | --- |
| Apodaca (2015) | BHSS Spanish version | very good | doubtful | very good | very good | doubtful | NA | NA | very good | very good | NA |
| Berger (2001) | BHSS | doubtful | NA | adequate | very good | NA | doubtful | NA | NA | very good | NA |
| Bunn (2007) | BHSS-32 | doubtful | NA | very good | very good | NA | NA | NA | very good | very good | NA |
| Chan (2019) | IARSS Southern India version | inadequate | doubtful | adequate | very good | doubtful | NA | NA | NA | very good | NA |
| Christopoulos  (2019) | IHSS3 | doubtful | NA | NA | very good | doubtful | NA | NA | NA | NA | NA |
| Cui (2021) | HAFSS | inadequate | doubtful | adequate | very good | NA | NA | NA | inadequate | NA | NA |
| Emlet (2005) | HASIP-13 | doubtful | NA | adequate | very good | inadequate | NA | NA | NA | very good | NA |

**Table 3 *(Continued)***

Methodological quality appraisal

| **Author**  **(Year)** | **PROM** | **PROM development** | **Content validity** | | **Construct validity** | | **Internal consistency** | | **Cross‐cultural validity/**  **measurement invariance** | | **Reliability** | | **Measure-ment error** | | **Criterion validity** | | **Hypothesis testing for construct validity** | | **Respon-siveness** | |  |
| --- | --- | --- | --- | --- | --- | --- | --- | --- | --- | --- | --- | --- | --- | --- | --- | --- | --- | --- | --- | --- | --- |
| Emlet (2007) | BHSS Spanish version 2 | inadequate | doubtful | | NA | | very good | | inadequate | | NA | | NA | | NA | | very good | | NA | |  |
| FIFE (2000) | HRSS | adequate | doubtful | | NA | | very good | | doubtful | | NA | | NA | | NA | | NA | | NA | |  |
| Franke (2010) | BHSS Spanish version 3 | inadequate | | doubtful | | adequate | | very good | | NA | | NA | | NA | | NA | | very good | | NA | |
| Garrido (2017) | IARSS Spanish version | adequate | | adequate | | adequate | | very good | | doubtful | | very good | | NA | | inadequate | | very good | | NA | |

**Table 3 *(Continued)***

Methodological quality appraisal

| **Author**  **(Year)** | **PROM** | **PROM development** | **Content validity** | | **Construct validity** | | **Internal consistency** | | **Cross‐cultural validity/**  **measurement invariance** | | **Reliability** | | **Measure-ment error** | | **Criterion validity** | | **Hypothesis testing for construct validity** | | **Respon-siveness** | |  |
| --- | --- | --- | --- | --- | --- | --- | --- | --- | --- | --- | --- | --- | --- | --- | --- | --- | --- | --- | --- | --- | --- |
| Geibel (2020) | IARSS Cambodia, the Dominican Republic, Uganda, Tanzania version | adequate | | adequate | | very good | | very good | | doubtful | | NA | | NA | | NA | | very good | | NA | |
| Han (2019) | EDS Chinese version | very good | | doubtful | | adequate | | very good | | NA | | very good | | NA | | inadequate | | very good | | NA | |
| Huang (2021) | BHSS Myanmar version | doubtful | | doubtful | | adequate | | very good | | inadequate | | doubtful | | NA | | NA | | very good | | NA | |

**Table 3 *(Continued)***

Methodological quality appraisal

| **Author**  **(Year)** | **PROM** | **PROM development** | **Content validity** | | **Construct validity** | | **Internal consistency** | | **Cross‐cultural validity/**  **measurement invariance** | | **Reliability** | | **Measure-ment error** | | **Criterion validity** | | **Hypothesis testing for construct validity** | | **Respon-siveness** | |  |
| --- | --- | --- | --- | --- | --- | --- | --- | --- | --- | --- | --- | --- | --- | --- | --- | --- | --- | --- | --- | --- | --- |
| Jeyaseelan (2013) | BHSS South Indian version | inadequate | | doubtful | | very good | | very good | | NA | | very good | | NA | | very good | | very good | | NA | |
| Jimenez (2010) | HFSS | very good | | doubtful | | adequate | | very good | | NA | | adequate | | NA | | NA | | very good | | NA | |
| Johnson (2016) | WHSS United States version | inadequate | | NA | | very good | | NA | | NA | | NA | | NA | | NA | | NA | | NA | |
| Kagiura (2020) | WHSS Japanese version | adequate | | doubtful | | very good | | very good | | NA | | NA | | NA | | NA | | very good | | NA | |
| Kalan (2013) | HASIP Iranian Version | very good | | very good | | very good | | very good | | NA | | very good | | NA | | NA | | NA | | NA | |

**Table 3 *(Continued)***

Methodological quality appraisal

| **Author**  **(Year)** | **PROM** | **PROM development** | **Content validity** | | **Construct validity** | | **Internal consistency** | | **Cross‐cultural validity/**  **measurement invariance** | | **Reliability** | | **Measure-ment error** | | **Criterion validity** | | **Hypothesis testing for construct validity** | | **Respon-siveness** |  |
| --- | --- | --- | --- | --- | --- | --- | --- | --- | --- | --- | --- | --- | --- | --- | --- | --- | --- | --- | --- | --- |
| Kalichman (2008) | IARSS | doubtful | | NA | | NA | | very good | | NA | | inadequate | | NA | | inadequate | | very good | NA | |
| Kamitani (2018) | WHSS United States version 2 | very good | | adequate | | adequate | | very good | | NA | | NA | | NA | | NA | | very good | NA | |
| Kingori (2013) | HASIP Kenyan version | doubtful | | doubtful | | adequate | | very good | | NA | | NA | | NA | | NA | | NA | NA | |
| Kipp (2015) | VRHRSS | doubtful | | NA | | adequate | | very good | | NA | | NA | | NA | | very good | | very good | NA | |
| Li (2010) | BHSS Chinese version | inadequate | | doubtful | | adequate | | very good | | NA | | doubtful | | NA | | inadequate | | NA | NA | |
| Li (2010) | HRSS and DS | very good | | doubtful | | adequate | | very good | | doubtful | | NA | | NA | | NA | | very good | NA | |

**Table 3 *(Continued)***

Methodological quality appraisal

| **Author**  **(Year)** | **PROM** | **PROM development** | **Content validity** | | **Construct validity** | | **Internal consistency** | | **Cross‐cultural validity/**  **measurement invariance** | | **Reliability** | | **Measure-ment error** | **Criterion validity** | **Hypothesis testing for construct validity** | **Respon-siveness** |
| --- | --- | --- | --- | --- | --- | --- | --- | --- | --- | --- | --- | --- | --- | --- | --- | --- |
| Lindberg (2014) | BHSS Swedish version | adequate | | doubtful | | very good | | very good | | doubtful | | NA | NA | NA | very good | NA |
| Luz (2020) | BHSS-12 Brazilian version | inadequate | | doubtful | | very good | | very good | | doubtful | | NA | NA | NA | very good | NA |
| Martin (2011) | IHSS Spanish and English version | doubtful | | NA | | very good | | very good | | NA | | NA | NA | NA | very good | NA |
| Molero (2013) | MSPD | adequate | | doubtful | | adequate | | very good | | adequate | | very good | NA | NA | NA | NA |
| Neufeld (2012) | HIV and ARSI | doubtful | | doubtful | | very good | | very good | | NA | | very good | NA | NA | NA | NA |

**Table 3 *(Continued)***

Methodological quality appraisal

| **Author**  **(Year)** | **PROM** | **PROM development** | **Content validity** | | **Construct validity** | | **Internal consistency** | | **Cross‐cultural validity/**  **measurement invariance** | | **Reliability** | | **Measure-ment error** | **Criterion validity** | **Hypothesis testing for construct validity** | **Respon-siveness** |
| --- | --- | --- | --- | --- | --- | --- | --- | --- | --- | --- | --- | --- | --- | --- | --- | --- |
| Öztürk (2020) | IARSS Turkish version | doubtful | | NA | | adequate | | very good | | NA | | NA | NA | NA | very good | NA |
| Phillips (2011) | ATIS | doubtful | | doubtful | | NA | | very good | | NA | | very good | NA | NA | NA | NA |
| Pourmarzi (2015) | HRSS Persian version | adequate | | adequate | | very good | | very good | | NA | | NA | NA | NA | very good | NA |
| Ranjit (2021) | WHSS Spanish version | very good | | doubtful | | adequate | | very good | | inadequate | | NA | NA | very good | NA | NA |
| Rao (2016) | CIBHSS | inadequate | | NA | | very good | | very good | | NA | | NA | NA | NA | NA | NA |
| Reinius (2017) | BHSS-12 Swedish version | very good | | doubtful | | adequate | | very good | | doubtful | | NA | NA | NA | very good | NA |

**Table 3 *(Continued)***

Methodological quality appraisal

| **Author**  **(Year)** | **PROM** | **PROM development** | **Content validity** | | **Construct validity** | | **Internal consistency** | | **Cross‐cultural validity/**  **measurement invariance** | | **Reliability** | | **Measure-ment error** | | **Criterion validity** | | **Hypothesis testing for construct validity** | | **Respon-siveness** | |  |
| --- | --- | --- | --- | --- | --- | --- | --- | --- | --- | --- | --- | --- | --- | --- | --- | --- | --- | --- | --- | --- | --- |
| Sayles (2008) | IHSS | doubtful | | doubtful | | adequate | | very good | | adequate | | NA | NA | | NA | | very good | | NA | |  |
| Stangl (2019) | IHSS2 | adequate | | adequate | | NA | | NA | | doubtful | | NA | NA | | NA | | very good | | NA | |  |
| Steward (2008) | HRS | inadequate | | NA | | very good | | very good | | NA | | NA | | NA | | inadequate | | very good | | NA | |
| Su et al. (2015) | BC-PLWH Chinese version | inadequate | | NA | | adequate | | very good | | NA | | very good | | NA | | NA | | very good | | NA | |
| Tsai (2013) | IARSS Uganda version | inadequate | | doubtful | | very good | | very good | | doubtful | | NA | | NA | | very good | | very good | | NA | |
| Visser (2008) | PSHS African version | adequate | | doubtful | | very good | | very good | | NA | | NA | | NA | | NA | | very good | | NA | |

**Table 3 *(Continued)***

Methodological quality appraisal

| **Author**  **(Year)** | **PROM** | **PROM development** | **Content validity** | | **Construct validity** | | **Internal consistency** | | **Cross‐cultural validity/**  **measurement invariance** | | **Reliability** | | **Measure-ment error** | | **Criterion validity** | | **Hypothesis testing for construct validity** | | **Respon-siveness** | |  |
| --- | --- | --- | --- | --- | --- | --- | --- | --- | --- | --- | --- | --- | --- | --- | --- | --- | --- | --- | --- | --- | --- |
| Xu (2018) | IHSS Chinese version | doubtful | | doubtful | | adequate | | very good | | NA | | very good | | NA | | very good | | NA | | NA | |
| Yu (2017) | BHSS Chinese version 2 | adequate | | adequate | | very good | | very good | | very good | | NA | | NA | | NA | | very good | | NA | |
| Zelaya (2012) | HSPS | adequate | | doubtful | | very good | | very good | | NA | | inadequate | | NA | | NA | | NA | | NA | |
