# Supplementary material for: Psychometric properties of stigma and discrimination measurement tools for persons living with HIV: a systematic review using the COSMIN methodology
Source: Syst Rev. 2024 Apr 27;13:115. doi: 10.1186/s13643-024-02535-y (PMC11055308; doi:10.1186/s13643-024-02535-y)
Supplement: Supplementary file 6 — Supplementary Material 6. [file 13643_2024_2535_MOESM6_ESM.docx]

**Table 4**

Measurement properties quality appraisal

| **Author**  **(Year)** | **PROM** | **Structural validity** | **Internal consistency** | **Cross‐cultural validity/**  **measurement invariance** | **Reliability** | **Measure-ment error** | **Criterion validity** | **Hypothesis testing for construct validity** | **Respon-siveness** |
| --- | --- | --- | --- | --- | --- | --- | --- | --- | --- |
| Apodaca (2015) | BHSS Spanish version | + | + | - | NA | NA | ? | + | NA |
| Berger (2001) | BHSS | ? | + | NA | ? | NA | NA | + | NA |
| Bunn (2007) | BHSS-32 | - | + | NA | NA | NA | ? | + | NA |
| Chan (2019) | IARSS Southern India version | ? | + | + | NA | NA | NA | + | NA |
| Christopoulos  (2019) | IHSS3 | NA | + | - | NA | NA | NA | NA | NA |

**Table 4 *(Continued)***

Measurement properties quality appraisal

| **Author**  **(Year)** | **PROM** | **Structural validity** | **Internal consistency** | **Cross‐cultural validity/**  **measurement invariance** | **Reliability** | **Measure-ment error** | **Criterion validity** | **Hypothesis testing for construct validity** | **Respon-siveness** |
| --- | --- | --- | --- | --- | --- | --- | --- | --- | --- |
| Cui (2021) | HAFSS | ? | + | NA | NA | NA | - | NA | NA |
| Emlet (2005) | HASIP-13 | ? | - | - | NA | NA | NA | + | NA |
| Emlet (2007) | BHSS Spanish version 2 | NA | + | - | NA | NA | NA | + | NA |
| FIFE (2000) | HRSS | NA | + | - | NA | NA | NA | NA | NA |
| Franke (2010) | BHSS Spanish version 2 | ? | - | NA | NA | NA | NA | + | NA |

**Table 4 *(Continued)***

Measurement properties quality appraisal

| **Author**  **(Year)** | **PROM** | **Structural validity** | **Internal consistency** | **Cross‐cultural validity/**  **measurement invariance** | **Reliability** | **Measure-ment error** | **Criterion validity** | **Hypothesis testing for construct validity** | **Respon-siveness** |
| --- | --- | --- | --- | --- | --- | --- | --- | --- | --- |
| Garrido (2017) | IARSS Spanish version | ? | + | - | + | NA | - | + | NA |
| Geibel (2020) | IARSS Cambodia, the Dominican Republic, Uganda, Tanzania version | + | + | - | NA | NA | NA | + | NA |

**Table 4 *(Continued)***

Measurement properties quality appraisal

| **Author**  **(Year)** | **PROM** | **Structural validity** | **Internal consistency** | **Cross‐cultural validity/**  **measurement invariance** | **Reliability** | **Measure-ment error** | **Criterion validity** | **Hypothesis testing for construct validity** | **Respon-siveness** |
| --- | --- | --- | --- | --- | --- | --- | --- | --- | --- |
| Han (2019) | EDS Chinese version | ? | + | NA | ? | NA | - | + | NA |
| Huang (2021) | BHSS Myanmar version | + | + | + | ? | NA | NA | + | NA |
| Jeyaseelan (2013) | BHSS South Indian version | + | - | NA | + | NA | ? | + | NA |
| Jimenez (2010) | HFSS | ? | + | NA | ? | NA | NA | + | NA |

**Table 4 *(Continued)***

Measurement properties quality appraisal

| **Author**  **(Year)** | **PROM** | **Structural validity** | **Internal consistency** | **Cross‐cultural validity/**  **measurement invariance** | **Reliability** | **Measure-ment error** | **Criterion validity** | **Hypothesis testing for construct validity** | **Respon-siveness** |
| --- | --- | --- | --- | --- | --- | --- | --- | --- | --- |
| Johnson (2016) | WHSS United States version | + | NA | NA | NA | NA | NA | NA | NA |
| Kagiura (2020) | WHSS Japanese version | + | + | NA | NA | NA | NA | + | NA |
| Kalan (2013) | HASIP Iranian Version | - | + | NA | + | NA | NA | NA | NA |
| Kalichman (2008) | IARSS | NA | + | NA | ? | NA | NA | + | NA |

**Table 4 *(Continued)***

Measurement properties quality appraisal

| **Author**  **(Year)** | **PROM** | **Structural validity** | **Internal consistency** | **Cross‐cultural validity/**  **measurement invariance** | **Reliability** | **Measure-ment error** | **Criterion validity** | **Hypothesis testing for construct validity** | **Respon-siveness** |
| --- | --- | --- | --- | --- | --- | --- | --- | --- | --- |
| Kamitani (2018) | WHSS United States version 2 | ? | + | NA | NA | NA | NA | + | NA |
| Kingori (2013) | HASIP Kenyan version | ? | - | NA | NA | NA | NA | NA | NA |
| Kipp (2015) | VRHRSS | ? | - | NA | NA | NA | ? | - | NA |
| Li (2010) | BHSS Chinese version | ? | - | NA | ? | NA | - | NA | NA |

**Table 4 *(Continued)***

Measurement properties quality appraisal

| **Author**  **(Year)** | **PROM** | **Structural validity** | **Internal consistency** | **Cross‐cultural validity/**  **measurement invariance** | **Reliability** | **Measure-ment error** | **Criterion validity** | **Hypothesis testing for construct validity** | **Respon-siveness** |
| --- | --- | --- | --- | --- | --- | --- | --- | --- | --- |
| Li (2010) | HRSS and DS | ? | + | - | NA | NA | NA | + | NA |
| Lindberg (2014) | BHSS Swedish version | + | - | - | NA | NA | NA | + | NA |
| Luz (2020) | BHSS-12 Brazilian version | + | - | - | NA | NA | NA | + | NA |
| Martin (2011) | IHSS Spanish and English version | + | + | NA | NA | NA | NA | + | NA |

**Table 4 *(Continued)***

Measurement properties quality appraisal

| **Author**  **(Year)** | **PROM** | **Structural validity** | **Internal consistency** | **Cross‐cultural validity/**  **measurement invariance** | **Reliability** | **Measure-ment error** | **Criterion validity** | **Hypothesis testing for construct validity** | **Respon-siveness** |
| --- | --- | --- | --- | --- | --- | --- | --- | --- | --- |
| Molero (2013) | MSPD | ? | + | + | - | NA | NA | NA | NA |
| Neufeld (2012) | HIV and ARSI | ? | + | NA | + | NA | NA | NA | NA |
| Öztürk (2020) | IARSS Turkish version | ? | + | NA | NA | NA | NA | + | NA |
| Phillips (2011) | ATIS | NA | - | NA | + | NA | NA | NA | NA |
| Pourmarzi (2015) | HRSS Persian version | + | + | NA | NA | NA | NA | + | NA |

**Table 4 *(Continued)***

Measurement properties quality appraisal

| **Author**  **(Year)** | **PROM** | **Structural validity** | **Internal consistency** | **Cross‐cultural validity/**  **measurement invariance** | **Reliability** | **Measure-ment error** | **Criterion validity** | **Hypothesis testing for construct validity** | **Respon-siveness** |
| --- | --- | --- | --- | --- | --- | --- | --- | --- | --- |
| Ranjit (2021) | WHSS Spanish version | ? | + | - | NA | NA | + | NA | NA |
| Rao (2016) | CIBHSS | + | + | NA | NA | NA | NA | NA | NA |
| Reinius (2017) | BHSS-12 Swedish version | ? | - | + | NA | NA | NA | + | NA |
| Sayles (2008) | IHSS | ? | + | - | NA | NA | NA | - | NA |
| Stangl (2019) | IHSS2 | NA | NA | - | NA | NA | NA | + | NA |

**Table 4 *(Continued)***

Measurement properties quality appraisal

| **Author**  **(Year)** | **PROM** | **Structural validity** | **Internal consistency** | **Cross‐cultural validity/**  **measurement invariance** | **Reliability** | **Measure-ment error** | **Criterion validity** | **Hypothesis testing for construct validity** | **Respon-siveness** |
| --- | --- | --- | --- | --- | --- | --- | --- | --- | --- |
| Steward (2008) | HRS | - | - | NA | NA | NA | - | + | NA |
| Su et al. (2015) | BC-PLWH Chinese version | ? | + | NA | - | NA | NA | + | NA |
| Tsai (2013) | IARSS Uganda version | - | - | - | NA | NA | NA | + | NA |
| Visser (2008) | PSHS African version | + | + | NA | NA | NA | NA | + | NA |
|  |  |  |  |  |  |  |  |  |  |

**Table 4 *(Continued)***

Measurement properties quality appraisal

| **Author**  **(Year)** | **PROM** | **Structural validity** | **Internal consistency** | **Cross‐cultural validity/**  **measurement invariance** | **Reliability** | **Measure-ment error** | **Criterion validity** | **Hypothesis testing for construct validity** | **Respon-siveness** |
| --- | --- | --- | --- | --- | --- | --- | --- | --- | --- |
| Xu (2018) | IHSS Chinese version | ? | + | NA | + | NA | - | NA | NA |
| Yu (2017) | BHSS Chinese version 2 | ? | + | - | NA | NA | NA | + | NA |
| Zelaya (2012) | HSPS | - | + | NA | ? | NA | NA | NA | NA |
