# Supplementary material for: Psychometric properties of stigma and discrimination measurement tools for persons living with HIV: a systematic review using the COSMIN methodology
Source: Syst Rev. 2024 Apr 27;13:115. doi: 10.1186/s13643-024-02535-y (PMC11055308; doi:10.1186/s13643-024-02535-y)
Supplement: Supplementary file 7 — Supplementary Material 7. [file 13643_2024_2535_MOESM7_ESM.docx]

**Table 5**

Evidence grading and recommendations

| **Recommended grade** | **PROM** | | **Content validity** | **Construct validity** | **Internal consistency** | **Cross‐cultural validity/**  **measurement invariance** | **Reliability** | **Measure-ment error** | **Criterion validity** | **Hypothesis testing for construct validity** | **Respon-siveness** |
| --- | --- | --- | --- | --- | --- | --- | --- | --- | --- | --- | --- |
| A | IARSS | IARSS | Low | NA | High | NA | Very Low | NA | NA | High | NA |
|  |  | IARSS Uganda version | Very Low | Moderate | High | NA | High | NA | NA | High | NA |
|  |  | IARSS Southern India version | Moderate | Moderate | High | Low | NA | NA | NA | High | NA |
|  |  | IARSS Turkish version | Moderate | High | Moderate | NA | Moderate | NA | NA | NA | NA |

**Table 5 *(Continued)***

Evidence grading and recommendations

| **Recommended grade** | **PROM** | | **Content validity** | **Construct validity** | **Internal consistency** | **Cross‐cultural validity/**  **measurement invariance** | **Reliability** | **Measure-ment error** | **Criterion validity** | **Hypothesis testing for construct validity** | **Respon-siveness** |
| --- | --- | --- | --- | --- | --- | --- | --- | --- | --- | --- | --- |
| A | IARSS | IARSS Cambodia, the Dominican Republic, Uganda, Tanzania version | High | High | High | Low | NA | NA | NA | High | NA |
|  |  | IARSS Spanish version | High | Moderate | High | Low | High | NA | Very Low | High | NA |
|  | IHSS | IHSS | Moderate | Moderate | High | Low | NA | NA | NA | Moderate | NA |
|  |  | IHSS *Chinese version* | Moderate | Moderate | High | NA | High | NA | High | NA | NA |

**Table 5 *(Continued)***

Evidence grading and recommendations

| **Recommended grade** | **PROM** | | **Content validity** | **Construct validity** | **Internal consistency** | **Cross‐cultural validity/**  **measurement invariance** | **Reliability** | **Measure-ment error** | **Criterion validity** | **Hypothesis testing for construct validity** | **Respon-siveness** |
| --- | --- | --- | --- | --- | --- | --- | --- | --- | --- | --- | --- |
| A | IHSS | IHSS *Spanish and English version* | Moderate | High | High | Low | NA | NA | NA | High | NA |
|  | WHSS | WHSS *Japanese version* | Moderate | High | High | NA | NA | NA | NA | High | NA |
|  |  | WHSS *Spanish version* | High | High | High | NA | NA | NA | NA | High | NA |
|  |  | WHSS United States version | Very Low | High | NA | NA | NA | NA | NA | NA | NA |

**Table 5 *(Continued)***

Evidence grading and recommendations

| **Recommended grade** | **PROM** | | **Content validity** | **Construct validity** | **Internal consistency** | **Cross‐cultural validity/**  **measurement invariance** | **Reliability** | **Measure-ment error** | **Criterion validity** | **Hypothesis testing for construct validity** | **Respon-siveness** |
| --- | --- | --- | --- | --- | --- | --- | --- | --- | --- | --- | --- |
| A | WHSS | WHSS *United States version 2* | High | Moderate | Moderate | NA | NA | NA | NA | Moderate | NA |
| B | ATIS | | Low | Moderate | High | NA | NA | NA | NA | High | NA |
|  | BHSS | BHSS | Low | Moderate | High | NA | Low | NA | NA | High | NA |
|  |  | BHSS Chinese version | Moderate | Moderate | High | NA | NA | NA | Very Low | NA | NA |
|  |  | BHSS Chinese version 2 | Moderate | High | High | NA | NA | NA | NA | High | NA |
|  |  | BHSS Myanmar version | Moderate | Moderate | High | Very Low | Low | NA | NA | High | NA |

**Table 5 *(Continued)***

Evidence grading and recommendations

| **Recommended grade** | **PROM** | | **Content validity** | **Construct validity** | **Internal consistency** | **Cross‐cultural validity/**  **measurement invariance** | **Reliability** | **Measure-ment error** | **Criterion validity** | **Hypothesis testing for construct validity** | **Respon-siveness** |
| --- | --- | --- | --- | --- | --- | --- | --- | --- | --- | --- | --- |
| B | BHSS | BHSS South Indian version | Moderate | High | High | NA | High | NA | High | High | NA |
|  |  | BHSS Spanish version | Moderate | High | High | Low | NA | NA | High | High | NA |
|  |  | BHSS Spanish version 2 | Low | NA | Low | Very Low | NA | NA | NA | Low | NA |
|  |  | BHSS Spanish version 3 | Moderate | Moderate | High | NA | NA | NA | NA | High | NA |
|  |  | BHSS Swedish version | Moderate | Moderate | High | Low | NA | NA | NA | High | NA |

**Table 5 *(Continued)***

Evidence grading and recommendations

| **Recommended grade** | **PROM** | | **Content validity** | **Construct validity** | **Internal consistency** | **Cross‐cultural validity/**  **measurement invariance** | **Reliability** | **Measure-ment error** | **Criterion validity** | **Hypothesis testing for construct validity** | **Respon-siveness** |
| --- | --- | --- | --- | --- | --- | --- | --- | --- | --- | --- | --- |
| B | BHSS | BHSS-12 Brazilian version | Moderate | High | High | Low | NA | NA | NA | High | NA |
|  |  | BHSS-12 Swedish version | Very Low | High | High | NA | NA | NA | NA | NA | NA |
|  |  | BHSS-32 | Low | High | High | NA | NA | NA | High | High | NA |
|  |  | CIBHSS | Moderate | Moderate | Moderate | Very Low | NA | NA | Moderate | NA | NA |
|  |  | HFSS | Moderate | Moderate | High | NA | Moderate | NA | NA | High | NA |
|  | HASIP | HASIP-13 | Low | Moderate | Moderate | Very Low | NA | NA | NA | Moderate | NA |
|  |  | HASIP *Iranian Version* | High | High | High | NA | Low | NA | NA | NA | NA |
|  |  | HASIP *Kenyan version* | Low | Moderate | High | NA | NA | NA | NA | NA | NA |

**Table 5 *(Continued)***

Evidence grading and recommendations

| **Recommended grade** | **PROM** | | **Content validity** | **Construct validity** | **Internal consistency** | **Cross‐cultural validity/**  **measurement invariance** | **Reliability** | **Measure-ment error** | **Criterion validity** | **Hypothesis testing for construct validity** | **Respon-siveness** |
| --- | --- | --- | --- | --- | --- | --- | --- | --- | --- | --- | --- |
| B | HRSS | HRSS | Moderate | NA | High | Low | NA | NA | NA | NA | NA |
|  |  | HRSS Persian version | Moderate | NA | Moderate | NA | Low | NA | NA | NA | NA |
|  | EDS *Chinese version* | | Moderate | Moderate | High | NA | NA | NA | Very Low | NA | NA |
|  | HAFSS | | Moderate | High | High | NA | NA | NA | NA | NA | NA |
|  | HIV and ARSI | | Moderate | Moderate | High | Moderate | Moderate | NA | NA | NA | NA |
|  | HRSS and DS | | Moderate | Moderate | High | NA | Low | NA | Very Low | High | NA |
|  | HSPS | | High | High | High | High | NA | NA | NA | High | NA |
|  | VRHRSS | | Low | Moderate | Moderate | NA | NA | NA | Moderate | Moderate | NA |
| C | BC-PLWH *Chinese version* | | Very Low | High | High | NA | NA | NA | Very Low | High | NA |
|  | HRS | | High | NA | NA | Low | NA | NA | NA | High | NA |
|  | IHSS *2* | | Low | Moderate | High | Moderate | NA | NA | NA | High | NA |
|  | IHSS *3* | | Very Low | NA | High | Low | NA | NA | NA | NA | NA |

**Table 5 *(Continued)***

Evidence grading and recommendations

| **Recommended grade** | **PROM** | **Content validity** | **Construct validity** | **Internal consistency** | **Cross‐cultural validity/**  **measurement invariance** | **Reliability** | **Measure-ment error** | **Criterion validity** | **Hypothesis testing for construct validity** | **Respon-siveness** |
| --- | --- | --- | --- | --- | --- | --- | --- | --- | --- | --- |
| C | MSPD | Very Low | High | High | NA | NA | NA | NA | High | NA |
|  | PSHS *African version* | Low | High | High | Low | NA | NA | NA | High | NA |

(A) PROMs with evidence for sufficient content validity (any level) AND at least low quality evidence for sufficient internal consistency;

(B) PROMs categorized not in A or C.

(C) PROMs with high quality evidence for an insufficient measurement property

PROMs categorized as 'A' can be recommended for use and results obtained with these PROMs can be trusted.

PROMs categorized as 'B' have potential to be recommended for use, but they require further research to assess the quality of these PROMs.

PROMs categorized as 'C' should not be recommended for use.
